# Supplementary material for: USP39 is essential for mammalian epithelial morphogenesis through upregulation of planar cell polarity components
Source: Commun Biol. 2022 Apr 19;5:378. doi: 10.1038/s42003-022-03254-7 (PMC9018712; doi:10.1038/s42003-022-03254-7)
Supplement: Supplementary file 2 — Supplementary Information [file 42003_2022_3254_MOESM2_ESM.pdf]

Title:

USP39 is essential for mammalian epithelial morphogenesis through upregulation of planar cell polarity components

Authors:

Chiharu Kimura-Yoshida, Kyoko Mochida, Shin-Ichiro Kanno, Isao Matsuo

This PDF file includes:

Figures S1 to S15
Supplementary figure legends
Tables S1 and S2
Supplementary Methods

Supplementary data 1 to 3 are separately uploaded.

Figure S1

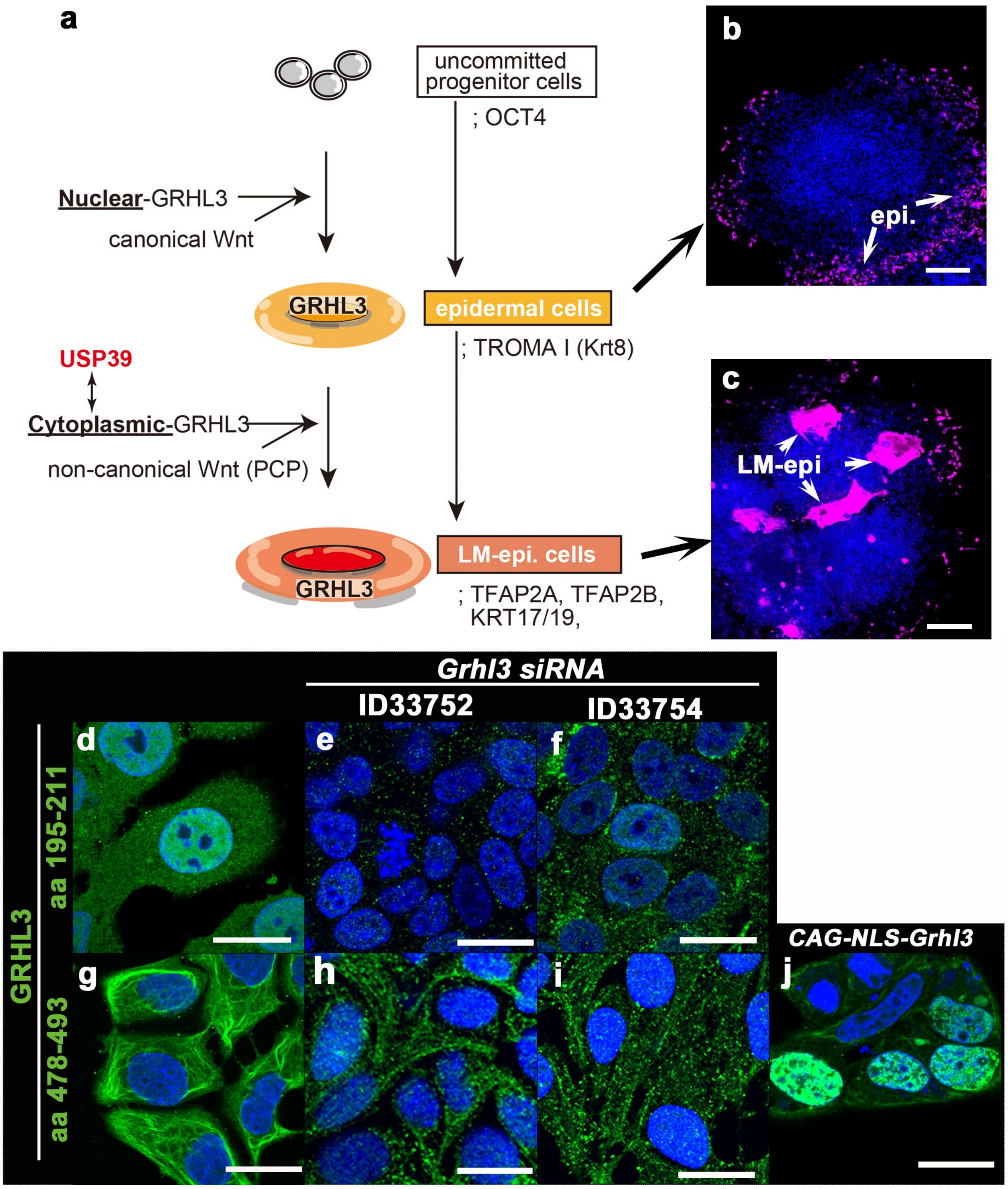
a
uncommitted progenitor cells
; OCT4
Nuclear-GRHL3
canonical Wnt
GRHL3
epidermal cells
; TROMA I (Krt8)
USP39
Cytoplasmic-GRHL3
non-canonical Wnt (PCP)
GRHL3
LM-epi. cells
; TFAP2A, TFAP2B, KRT17/19,
b
epi.
c
LM-epi
Grhl3 siRNA
ID33752
ID33754
GRHL3
aa 195-211
aa 478-493
d
e
f
g
h
i
j
CAG-NLS-Grhl3

Figure S2

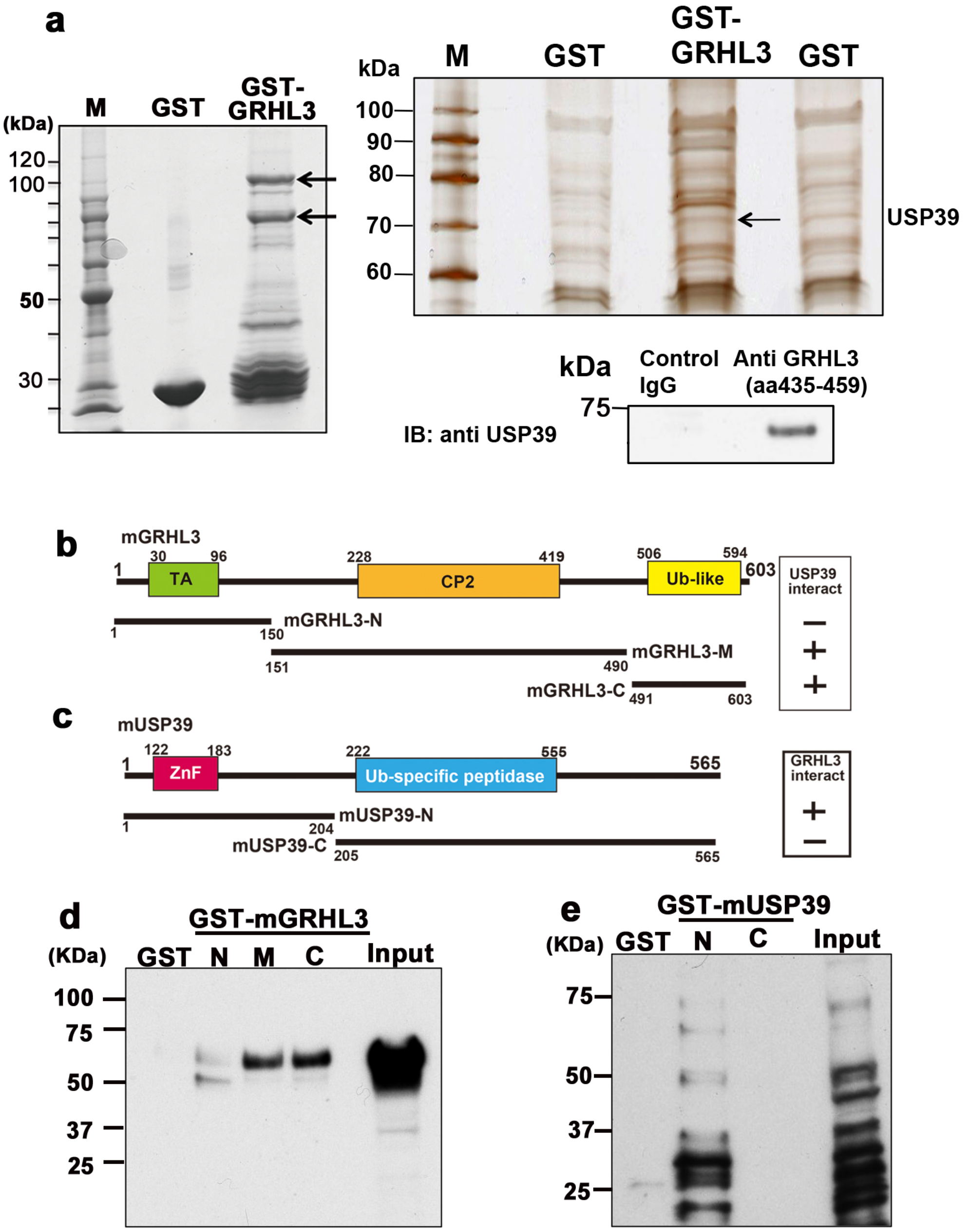
a
(kDa)
M
GST
GST-
GRHL3
120
100
50
30
kDa
100
90
80
70
60
USP39
kDa
Control
IgG
Anti GRHL3
(aa435-459)
75
IB: anti USP39
b
mGRHL3
1
30
96
TA
228
419
CP2
506
594
Ub-like
603
USP39
interact
mGRHL3-N
150
151
490
mGRHL3-M
mGRHL3-C
491
603
−
+
+
c
mUSP39
122
183
ZnF
222
555
Ub-specific peptidase
565
GRHL3
interact
mUSP39-N
204
205
mUSP39-C
+
−
d
GST-mGRHL3
(KDa)
GST
N
M
C
Input
100
75
50
37
25
e
GST-mUSP39
(KDa)
GST
N
C
Input
75
50
37
25

Figure S3

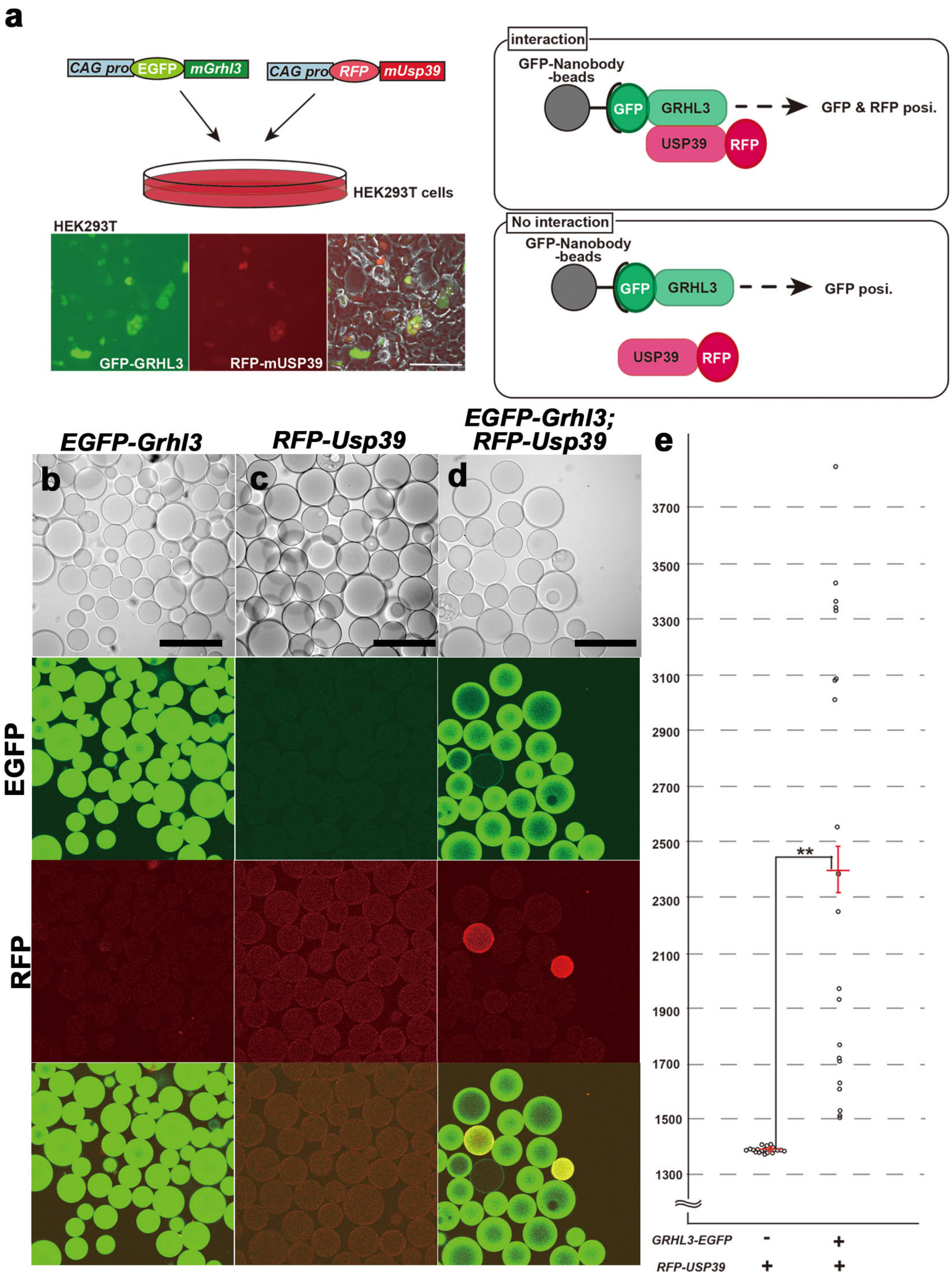
a
CAG pro
EGFP
mGrhl3
CAG pro
RFP
mUsp39
HEK293T cells
HEK293T
GFP-GRHL3
RFP-mUSP39
interaction
GFP-Nanobody -beads
GFP
GRHL3
USP39
RFP
GFP & RFP posi.
No interaction
GFP-Nanobody -beads
GFP
GRHL3
GFP posi.
USP39
RFP
EGFP-Grhl3
RFP-Usp39
EGFP-Grhl3; RFP-Usp39
b
c
d
EGFP
RFP
e
3700
3500
3300
3100
2900
2700
2500
2300
2100
1900
1700
1500
1300
**
GRHL3-EGFP - +
RFP-USP39 + +

Figure S4

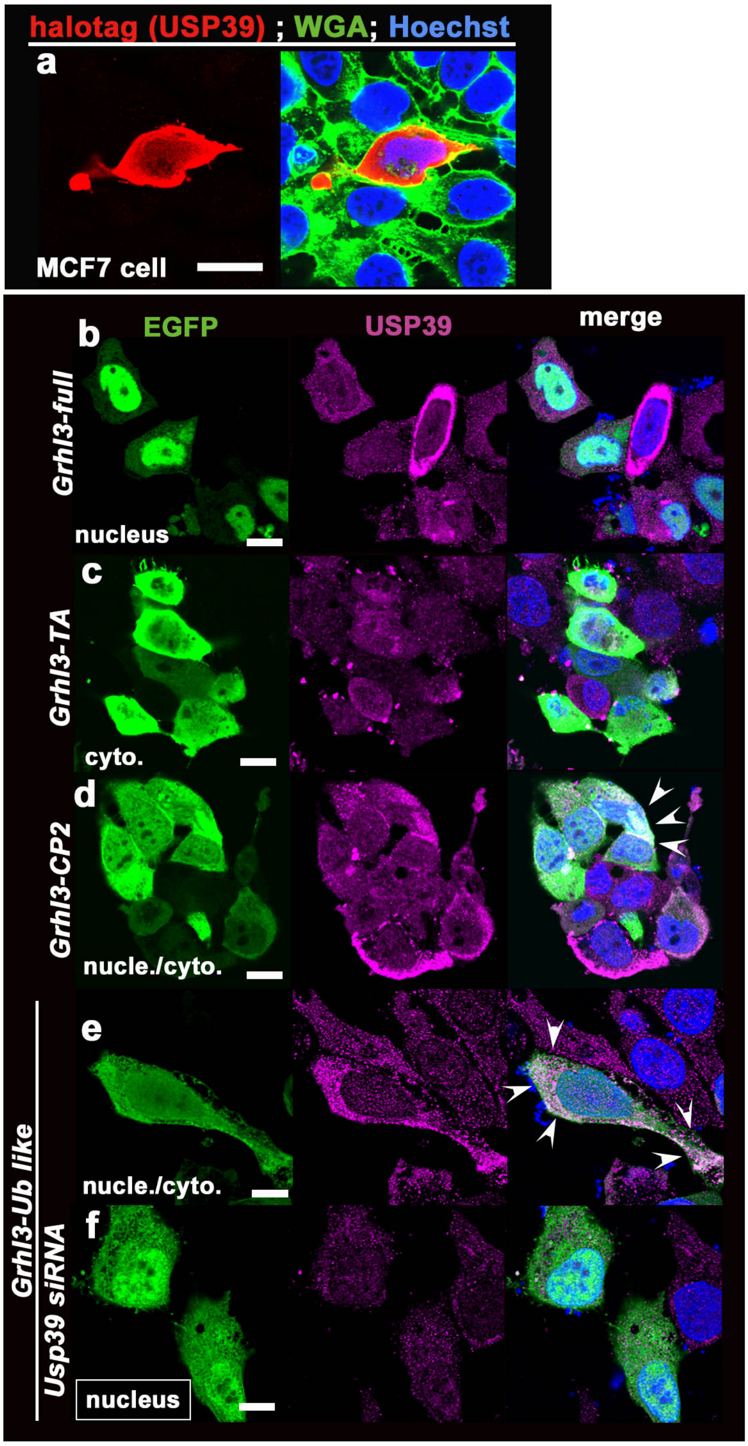
halotag (USP39) ; WGA; Hoechst
a
MCF7 cell
b
EGFP
USP39
merge
Grhl3-full
nucleus
c
Grhl3-TA
cyto.
d
Grhl3-CP2
nucle./cyto.
e
Grhl3-Ub like
nucle./cyto.
f
Usp39 siRNA
nucleus

Figure S5

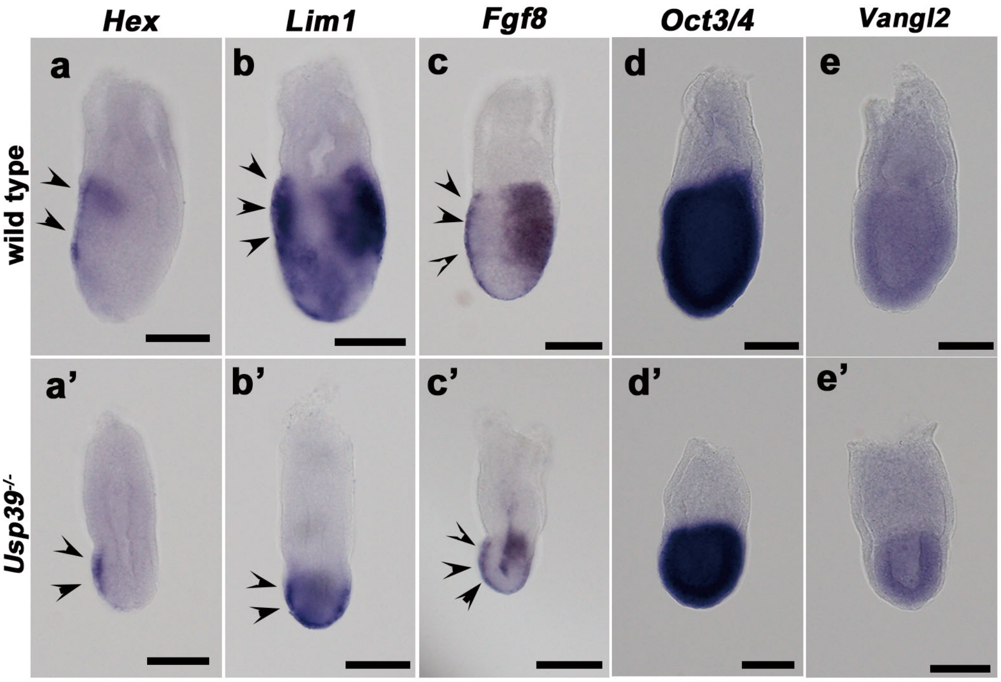

Figure S6

	#7 wild type			#8 wild type			#11 wild type			#12 wild type		
	vertical	horizontal	ratio	vertical	horizontal	ratio	vertical	horizontal	ratio	vertical	horizontal	ratio
#1	1.4881	0.6836	2.17685781	1.2702	0.3116	4.07637997	0.8679	0.6267	1.38487315	1.2149	0.5657	2.14760474
#2	1.2653	0.4158	3.04304954	1.647	0.2766	5.95444685	1.27	0.3263	3.89212381	1.3684	0.4763	2.87297921
#3	1.2617	0.7076	1.78306953	1.1937	0.4175	2.85916168	1.1814	0.511	2.31193738	0.8929	0.5667	1.5756132
#4	1.2112	0.4294	2.82068002	1.0276	0.441	2.33015873	1.1433	0.5992	1.90804406	1.1865	0.5647	2.10111564
#5	1.4582	0.4527	3.22111774	1.2171	0.3763	3.23438746	1.4108	0.493	2.86166329	1.3759	0.6389	2.15354516
#6	1.1533	0.6589	1.75034148	0.8665	0.6129	1.4137706	1.3768	0.6535	2.10680949	1.1061	0.4439	2.49177743
#7	0.9147	0.521	1.75566219	1.4231	0.5172	2.75154679	1.1081	0.6731	1.64626356	1.342	0.5055	2.65479723
#8	1.2743	0.4351	2.92875201	1.1565	0.4957	2.33306435	1.2423	0.3842	3.23347215	1.2548	0.6882	1.82330718
#9	1.5938	0.4872	3.27134647	1.0497	0.6121	1.71491586	1.3989	0.5221	2.67937177	0.954	0.5969	1.59825766
#10	1.1717	0.6099	1.92113461	1.077	0.6607	1.6300893	1.1624	0.6267	1.85479496	1.4845	0.3961	3.74779096
#11	1.3665	0.559	2.44454383	1.2895	0.4961	2.59927434	1.1812	0.6778	1.74269696	1.0678	0.497	2.14849095
#12	1.3189	0.5386	2.44875603	1.2433	0.5234	2.37542988	1.0939	0.6352	1.72213476	1.0863	0.4668	2.32712082
#13	1.4362	0.354	4.05706215	1.1197	0.4234	2.64454417	1.9402	0.55	3.52763636	0.9892	0.6453	1.53293042
#14	0.9498	0.3369	2.81923419	0.9116	0.5023	1.81485168	0.1545	0.5319	0.29046813	0.9652	0.5766	1.67395075
#15	1.2685	0.4704	2.69664116	0.5371	0.5556	0.96670266	1.0325	0.5832	1.77040466	1.7868	0.4394	4.06645426
#16	1.055	0.4564	2.3115688	Av.		2.57991496	1.0364	0.5951	1.74155604	1.6779	0.4154	4.03923929
#17	0.9052	0.4266	2.12189405				1.107	0.6052	1.82914739	1.2664	0.6615	1.91443689
#18	0.8921	0.5185	1.72054002				1.2279	0.3186	3.85404896	0.8877	0.6476	1.37075355
#19	1.0862	0.5936	1.82985175				0.8984	0.4481	2.00490962	Av.		2.34667585
#20	1.8085	0.498	3.6315261				1.0152	0.5116	1.98436278			
	Av.		2.53768147				Av.		2.21733596			

	#6 Usp39 KO			#9 Usp39 KO			#10 Usp39 KO			#17 Usp39 KO		
	vertical	horizontal	ratio	vertical	horizontal	ratio	vertical	horizontal	ratio	vertical	horizontal	ratio
#1	0.7436	0.6405	1.16096799	0.8625	0.6706	1.28616165	0.7975	0.8667	0.92015692	1.2813	0.4825	2.65554404
#2	0.6438	0.5052	1.27434679	0.639	0.9279	0.68865179	0.8786	0.6045	1.45343259	0.9734	0.507	1.9199211
#3	1.2654	0.4947	2.55791389	0.9315	0.4538	2.05266637	0.7258	0.4146	1.75060299	1.0495	0.7182	1.46129212
#4	0.6011	0.6879	0.87381887	0.7553	0.437	1.72837529	0.7096	0.6351	1.11730436	0.9819	0.538	1.82509294
#5	0.812	0.5152	1.57608696	1.1236	0.691	1.6260492	0.7955	0.8458	0.94052968	1.28	0.5941	2.15451944
#6	0.8051	0.5733	1.40432583	0.9261	0.6426	1.44117647	1.2493	0.7039	1.77482597	0.9998	0.4322	2.31328089
#7	1.2352	0.3969	3.11211892	0.763	0.4102	1.86006826	1.2779	0.7307	1.74887095	1.1386	0.5688	2.00175809
#8	0.9525	0.4942	1.92735735	0.781	0.4076	1.91609421	0.7691	0.6392	1.20322278	1.0676	0.4947	2.1580756
#9	1.004	0.6208	1.61726804	1.2176	0.674	1.80652819	0.9254	0.7182	1.28849903	1.0268	0.5381	1.9081955
#10	0.8915	0.7898	1.12876678	0.6731	0.7389	0.91094871	0.8084	0.5557	1.45474177	0.9916	0.6306	1.57247066
#11	1.1106	0.6491	1.71098444	0.8046	0.8632	0.93211307	0.7273	0.8845	0.82227247	0.9198	0.6234	1.47545717
#12	1.0602	0.7251	1.46214315	1.152	0.865	1.33179191	0.8651	0.5494	1.57462687	0.7322	0.5479	1.33637525
#13	0.9069	0.8571	1.05810291	0.5406	0.449	1.20400891	0.9713	0.5306	1.83056917	1.0181	0.6879	1.48001163
#14	0.8936	0.703	1.27112376	0.7989	0.7604	1.05063125	0.8345	0.6209	1.34401675	0.761	0.6754	1.12673971
#15	0.5381	0.7234	0.74384849	1.0718	0.6746	1.58879336	0.8283	0.7509	1.10307631	1.3894	0.5469	2.54050101
#16	0.8229	0.5606	1.46789154	0.5023	0.7373	0.6812695	0.4658	0.5012	0.92936951	0.795	0.5618	1.41509434
#17	0.8137	0.7297	1.1151158	0.9917	0.7196	1.37812674	0.9264	0.7628	1.21447299	1.0188	0.5887	1.73059283
#18	1.153	0.4851	2.37682952	0.939	0.6433	1.45966112	0.8291	0.4977	1.66586297	1.0878	0.6942	1.56698358
#19	1.2024	0.7862	1.52938184	1.1762	0.753	1.56201859	0.9605	0.7339	1.30876141	0.688	0.4942	1.39214893
#20	0.9547	0.5759	1.65775308	Av.		1.39500708	0.7598	0.6837	1.11130613	1.0005	0.5164	1.93745159
	Av.		1.5513073				Av.		1.32782608	Av.		1.79857532

	#13 Usp39 KO			#14 Usp39 KO		
	vertical	horizontal	ratio	vertical	horizontal	ratio
#1	0.9227	0.5657	1.63107654	1.2778	0.7083	1.80403784
#2	1.2398	0.806	1.5382134	1.0137	0.5998	1.69006335
#3	0.6888	0.6267	1.09909047	1.2146	0.7234	1.67901576
#4	0.7638	0.4966	1.5380588	0.8562	0.6174	1.38678328
#5	1.0064	0.7427	1.35505588	0.6792	0.6292	1.07946599
#6	0.688	0.7373	0.93313441	0.9777	0.46	2.12543478
#7	0.7067	0.6882	1.02688172	1.3963	0.7149	1.9531403
#8	1.3551	0.8485	1.59705362	1.538	0.4792	3.20951586
#9	0.7234	0.8757	0.82608199	1.2543	0.5406	2.32019978
#10	0.8894	0.7515	1.18349967	1.503	0.883	1.70215176
#11	1.0165	0.4933	2.0606122	0.9126	0.7029	1.29833547
#12	0.9417	0.419	2.24749403	1.1618	0.7652	1.51829587
#13	0.8095	0.7559	1.07090885	0.8887	0.731	1.21573187
#14	0.8219	0.5386	1.52599332	1.1271	0.6601	1.70746857
#15	0.8616	0.6117	1.40853359	1.2094	0.6248	1.93565941
#16	1.2	0.4617	2.59909032	1.1659	0.6605	1.7651779
#17	1.2084	0.5391	2.24151363	1.2318	0.4937	2.49503747
#18	0.8738	0.5398	1.61874768	1.0138	0.454	2.23303965
#19	0.8733	0.4086	2.13729809	Av.		1.83991972
#20	1.2142	0.6192	1.96091731			
	Av.		1.57996278			

Figure S7

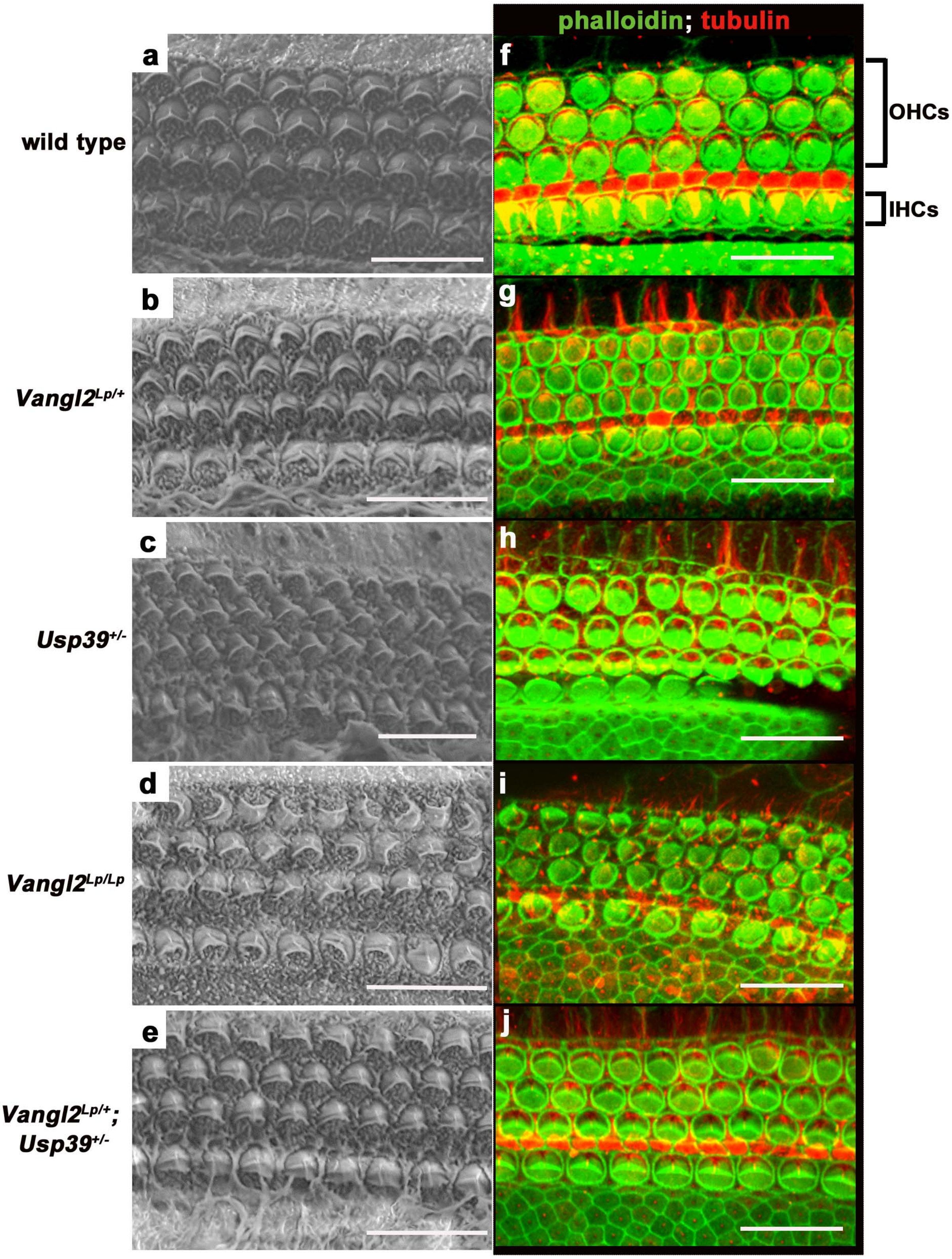
phalloidin; tubulin
a
b
c
d
e
f
g
h
i
j
wild type
Vangl2Lp/+
Usp39+/-
Vangl2Lp/Lp
Vangl2Lp/+;
Usp39+/-
OHCs
IHCs

Figure S8

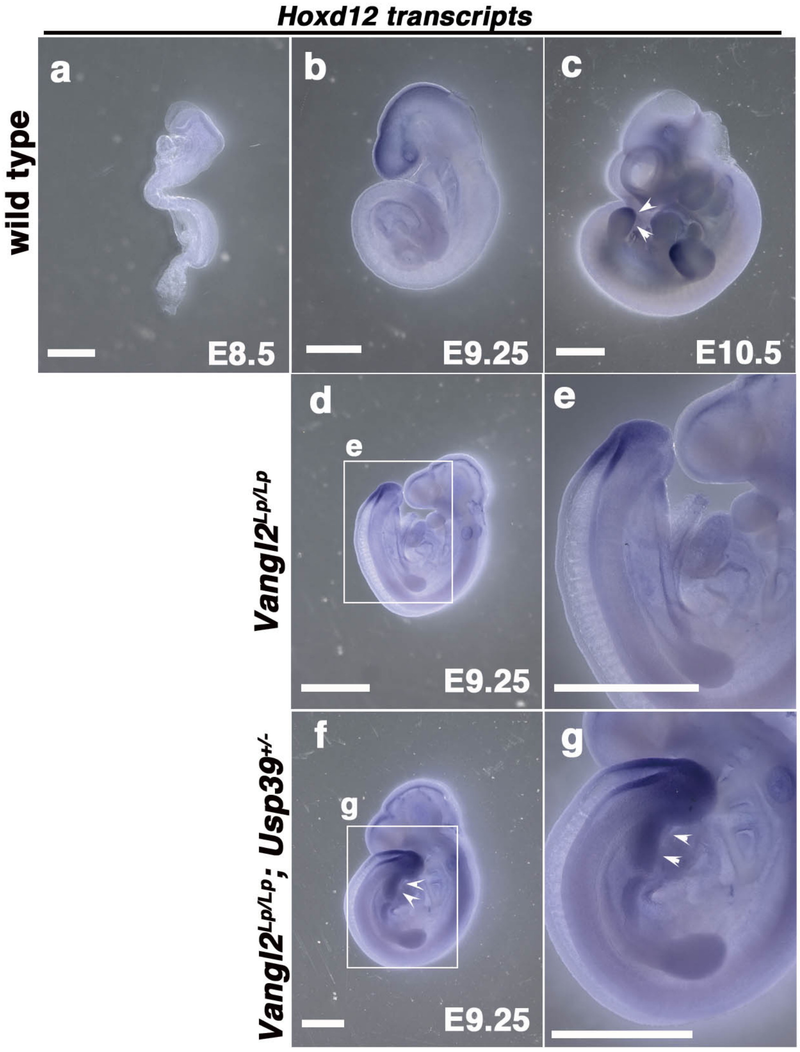
Hoxd12 transcripts
wild type
Vangl2Lp/Lp
Vangl2Lp/Lp; Usp39+/-
a
b
c
d
e
f
g
E8.5
E9.25
E10.5
E9.25
E9.25

Figure S9

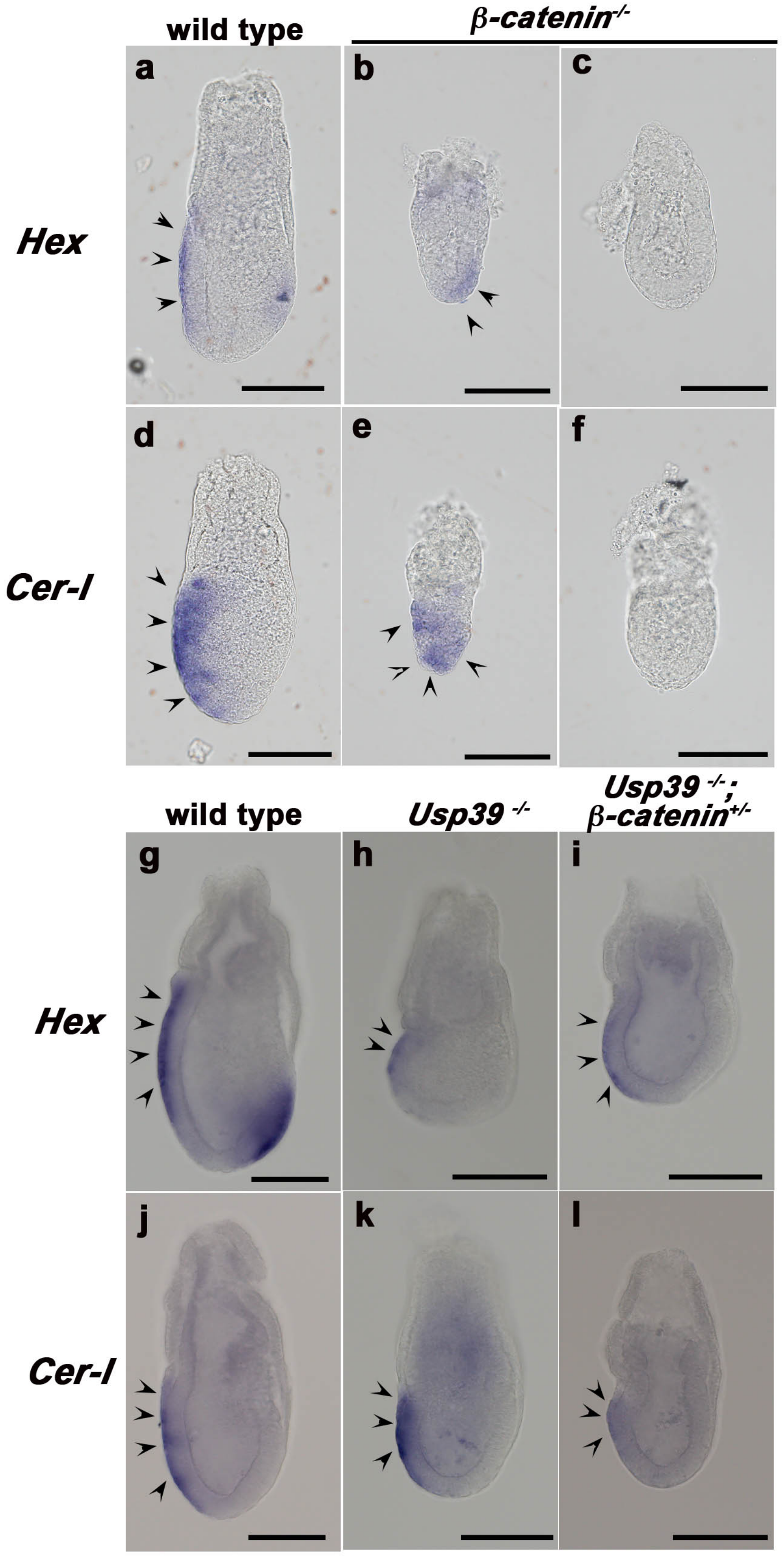
wild type
β-catenin-/-
a
b
c
Hex
d
e
f
Cer-l
wild type
Usp39 -/-
Usp39 -/-;
β-catenin+/-
g
h
i
Hex
j
k
l
Cer-l

Figure S10

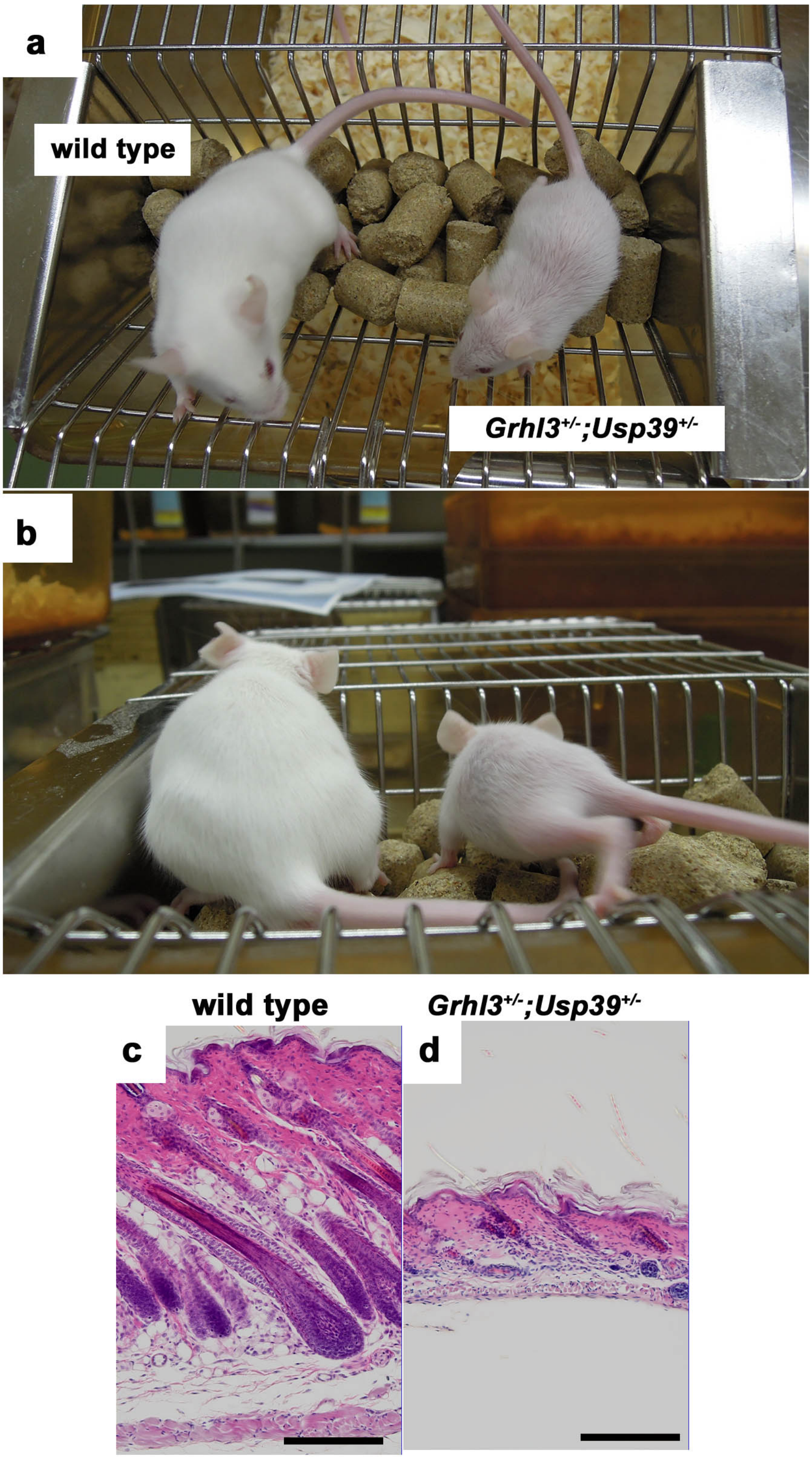

Figure S11

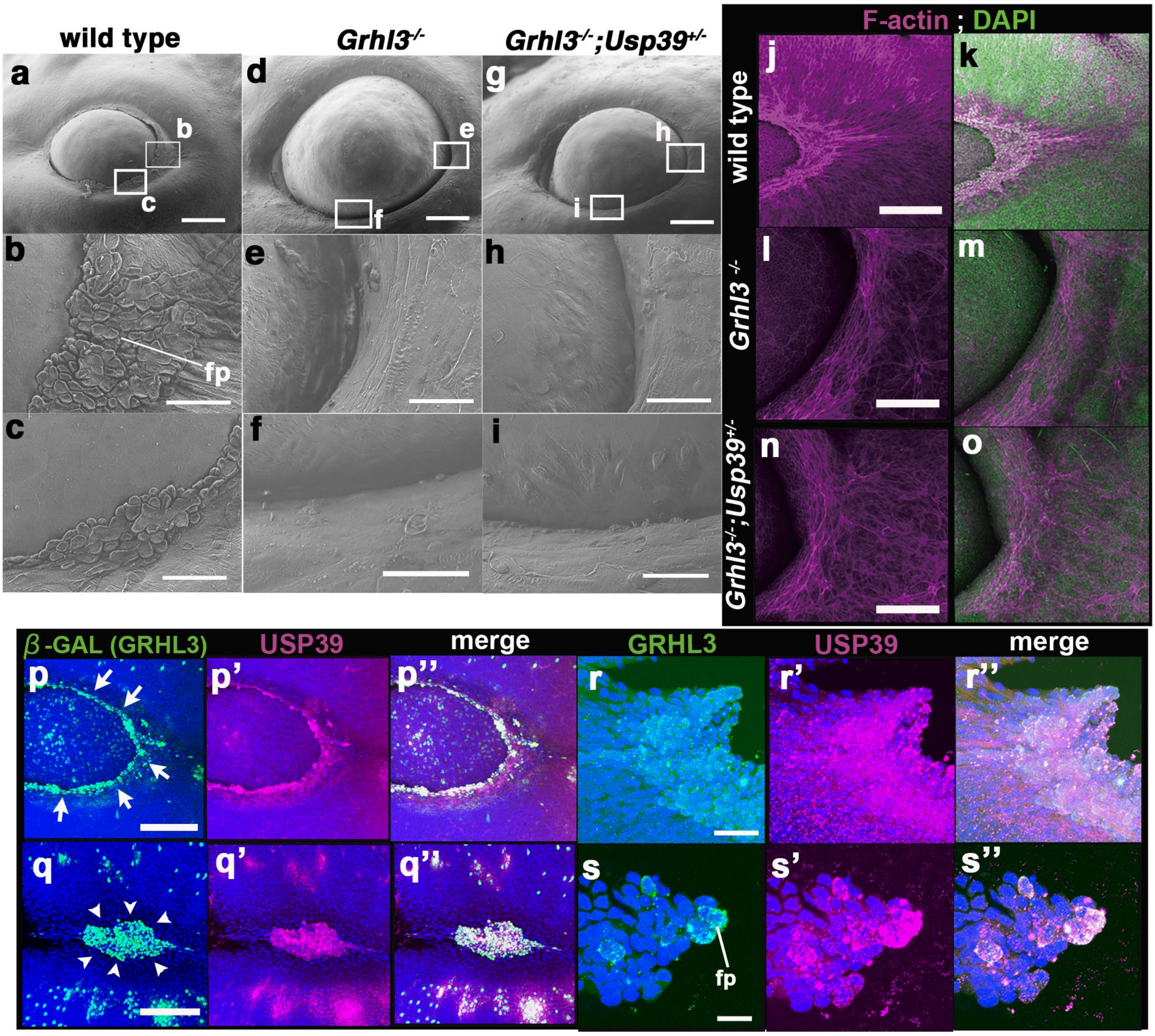

wild type
Grhl3-/-
Grhl3-/-;Usp39+/-
a
b
c
d
e
f
g
h
i
fp
F-actin ; DAPI
j
k
l
m
n
o
wild type
Grhl3 -/-
Grhl3-/-;Usp39+/-
β-GAL (GRHL3)
USP39
merge
GRHL3
USP39
merge
p
p'
p''
q
q'
q''
r
r'
r''
s
s'
s''
fp

Figure S12

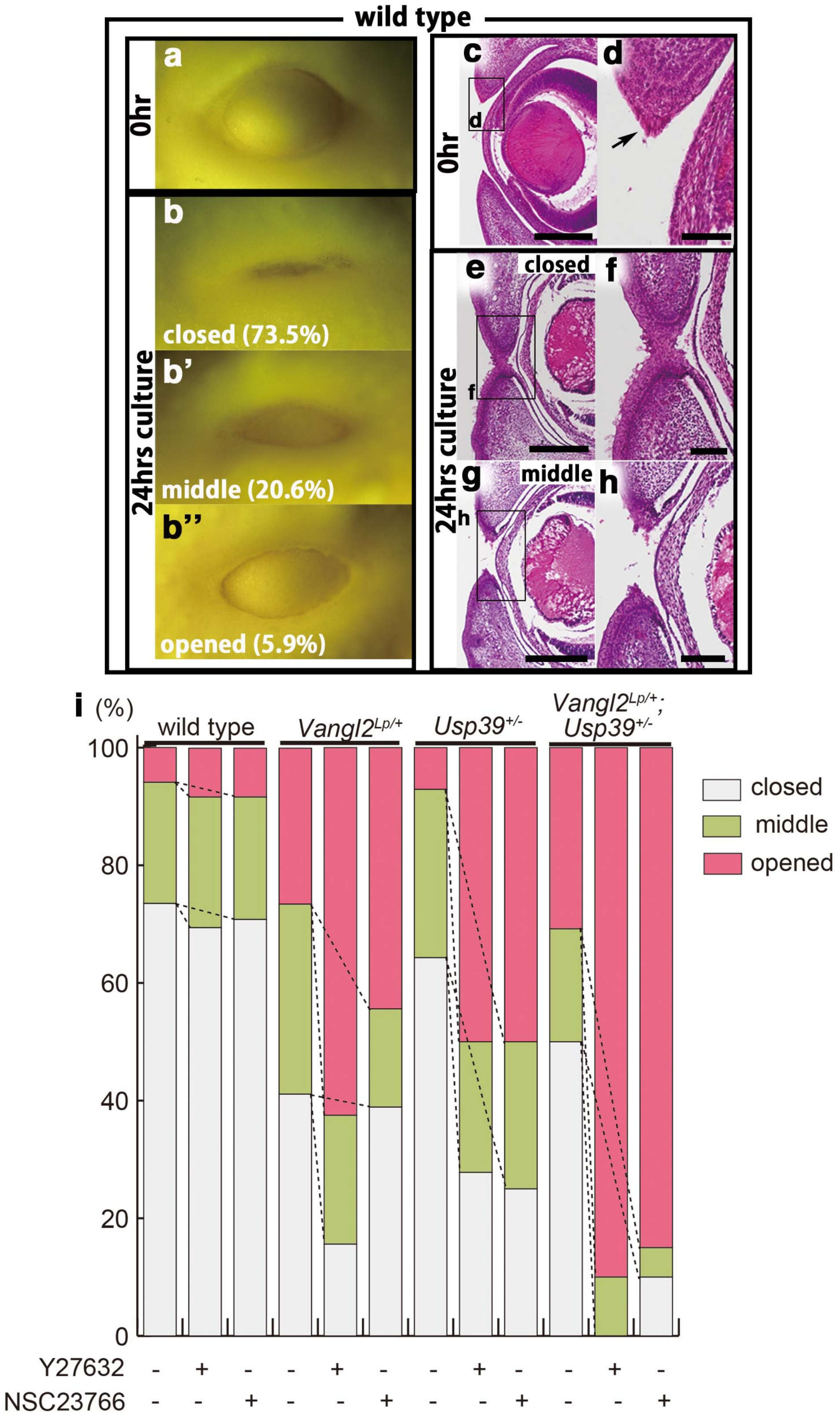
wild type
a
0hr
b
closed (73.5%)
b'
middle (20.6%)
b''
opened (5.9%)
24hrs culture
c
d
0hr
e
closed
f
g
middle
h
24hrs culture
i
(%)
wild type
Vangl2Lp/+
Usp39+/-
Vangl2Lp/+;
Usp39+/-
100
80
60
40
20
0
closed
middle
opened
Y27632 - + - - + - - + - - + -
NSC23766 - - + - - + - - + - - +

Figure S13

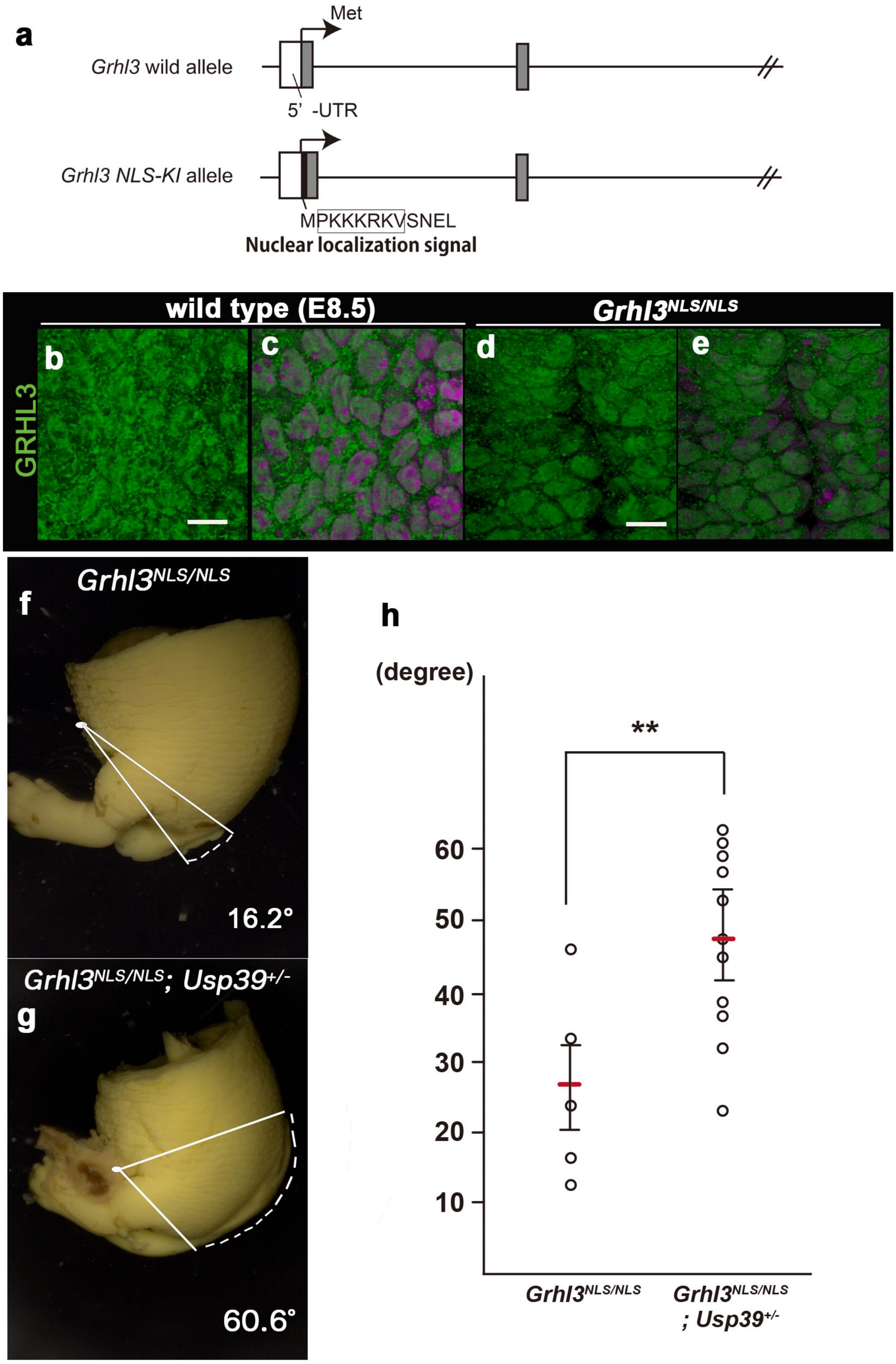
a
Met
Grhl3 wild allele
5' -UTR
Grhl3 NLS-KI allele
MPKKKRKVSNEL
Nuclear localization signal
wild type (E8.5)
Grhl3NLS/NLS
b
c
d
e
GRHL3
f
Grhl3NLS/NLS
16.2°
Grhl3NLS/NLS; Usp39+/-
g
60.6°
h
(degree)
**
60
50
40
30
20
10
Grhl3NLS/NLS
Grhl3NLS/NLS
; Usp39+/-

Figure S14

a

Name	Fold change	*P value*
down (>-2 fold)		
Dact1	- 4.6092	6.53E-12
Ror2	- 2.3804	4.55E-05
Prickle1	- 2.2533	1.23E-06
Fzd2	- 2.2474	1.46E-09

b

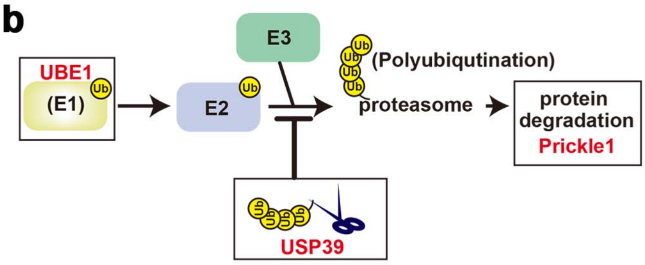

c

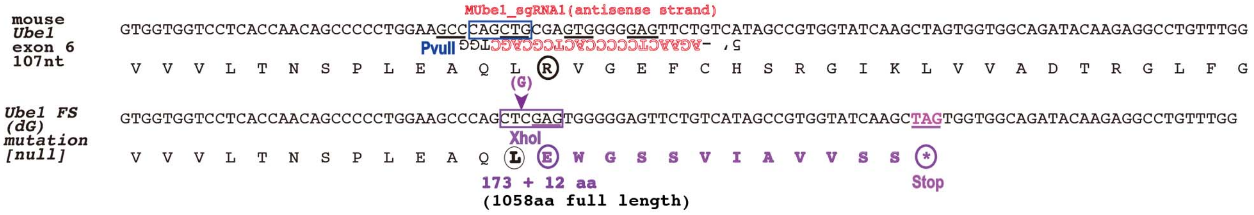

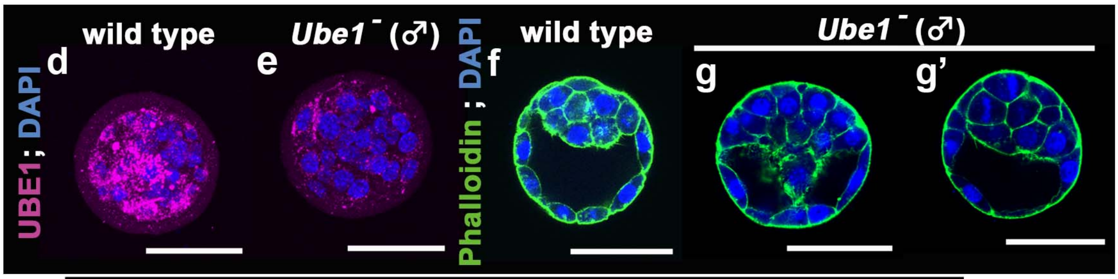

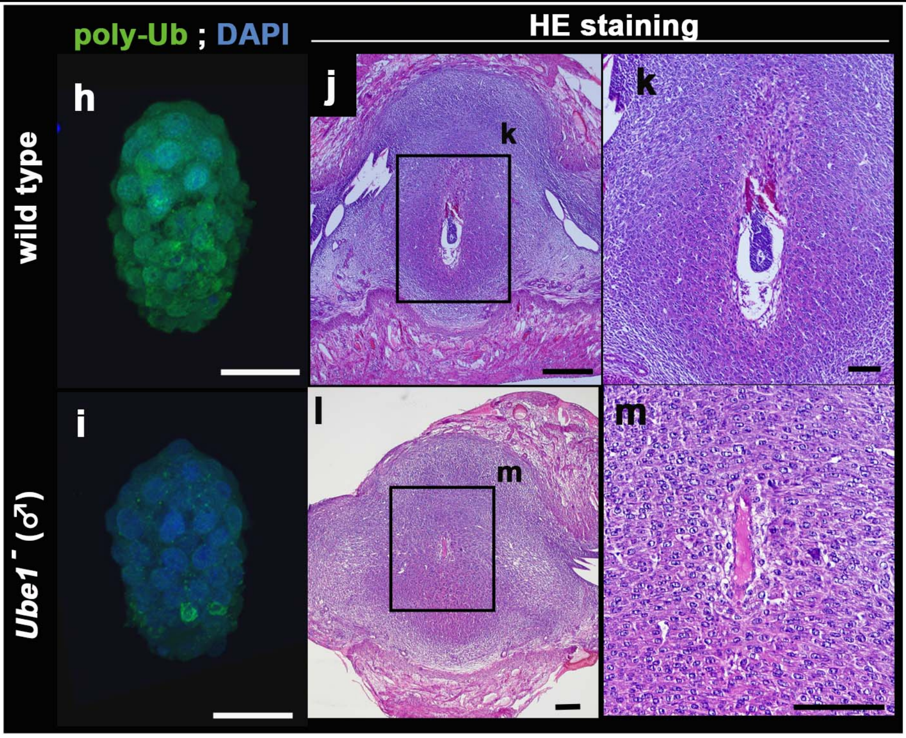

n *Usp39+/-;Ube1- (F) x Usp39+/- (M)*

	Wt	*Usp39*+/-	*Ube1*+/-	*Usp39*+/-; *Ube1*+/-	*Usp39*-/-	*Usp39*-/-; *Ube1*+/-	Absorbed	total
	16	37	10	22	10	4	41	140 (11 litters)
Frequencies (%)	11.4	26.4	7.2	15.7	7.1	2.9	29.3	100
Predicted (%)	12.5	25	6.25	12.5	12.5	6.25	25	100

Figure S15

Fig.S2a

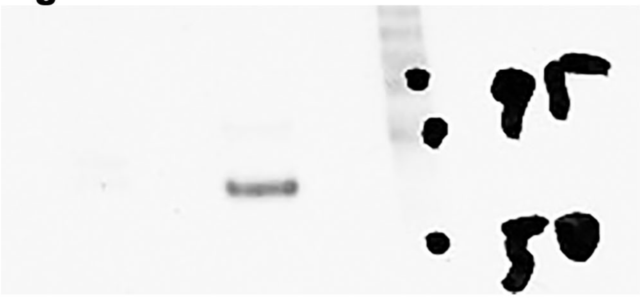

Fig.S2d

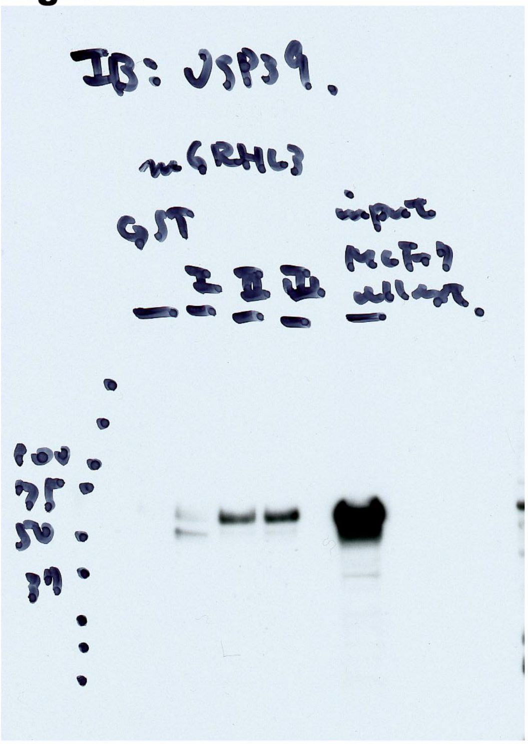

Fig.S2e

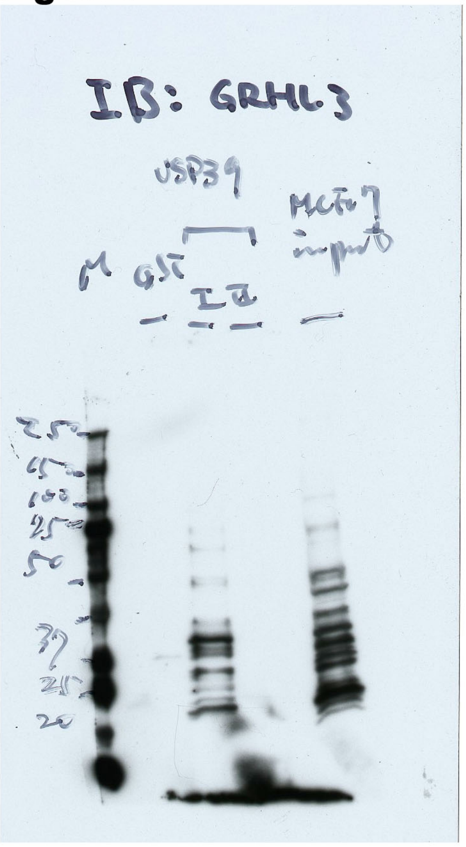

Uncropped blots of Fig.S2

Supplementary Figure legends

Figure S1. Induction of large and mature epidermal cells from embryoid bodies by GRHL3 and subcellular localization of GRHL3 in MCF7 cells.
(a–c) Schematic diagrams for molecular events during the induction of large and mature (LM)-epidermal cells from embryonic stem (ES) cell–derived embryoid bodies (EBs) by *Grhl3* cDNA overexpression (a–c). Grainyhead-like 3 (GRHL3) protein initially promotes the differentiation of uncommitted progenitor cells into epidermal cells by cooperating with canonical Wnt in the nucleus (b; epi). Subsequently, in induced epidermal cells, GRHL3 protein translocates from the nucleus to cytoplasm and cooperates with non-canonical Wnt (planar cell polarity [PCP] pathway) to generate LM-epidermal cells (c). *Grhl3* expression can induce LM-epidermal cells that are enriched in actomyosin networks. We hypothesize that the induction of LM-epidermal cells needs the cytoplasmic localization of GRHL3 protein from the nucleus that is mediated by ubiquitin-specific protease 39 (USP39). The USP39 positively modulates non-canonical Wnt signaling through expression of PCP components, including cytoplasmic localization of GRHL3, and, consequently, provides LM-epidermal cells with sufficient mechanical properties for epithelial morphogenesis such as a change in cell shape. (d–j) Immunohistochemistry of GRHL3 in MCF7 cells using two different antibodies against GRHL3 peptides; synthetic peptides corresponding to from the 195th to 211th aa (d–f) and from the 478th to 493th aa (g–j). GRHL3 antibodies (green) and 4,6'-diamidino-2-phenylindole (DAPI; blue). GRHL3 protein expression with antibodies against aa 478–493 was predominantly found in the cytoplasm (g); using antibody against aa 195–211, GRHL3 was observed in the nucleus (d). GRHL3 protein expression with an antibody against aa 478–493 was predominantly observed in the nucleus by transfecting *Grhl3* cDNA fused with nuclear localization signaling (NLS) at the N-terminal (j) [1]. *Grhl3* small interfering *(*si)RNA (ID33752 and ID33754) reduced endogenous GRHL3 expression (e,f,h,i). Abbreviations: epi, epidermal cells; LM-epi, large and mature epidermal cells; Scale bars represent 20 μm (b–j).

Figure S2. Identification of USP39 as a GRHL3-binding protein and its interaction.
(a) Putative grainyhead-like 3 (GRHL3)–binding proteins identified by affinity purification using glutathione *S*-transferase (GST)-GRHL3 beads and mass spectrometry. The top left panel indicates GST-GRHL3 fused proteins stained with Coomassie Brilliant Blue dye (arrows). The right panel indicates a silver stain of affinity-purified proteins using GST and GST-GRHL3 beads, and protein bands identified by nanoscale liquid chromatography coupled to a tandem mass spectrometry (nanoLC/MS/MS) system (arrows). The bottom right panel indicates immunoprecipitation with antibody against GRHL3 showing that ubiquitin-specific protease 39 (USP39) co-immunoprecipitated with GRHL3 in MCF7 cell extracts in which no transgenes were transfected. (b–e) Domain-mapping for the physical interaction between GRHL3 and USP39. Three domains (mGRHL3-N, -M, and -C) of GRHL3 and two domains (mUSP39-N and -C) of USP39 tagged with a GST fusion protein are schematically shown (b,c). GST-tagged middle or C-terminals of GRHL3 are able to bind directly with USP39 (d). Western blotting with an antibody against USP39 (abcam ab131332) (d). The N-terminal domain of USP39 binds GRHL3. Western blotting with an antibody against GRHL3 (Santa Cruz sc-398838) (e).

Figure S3. Interaction of GRHL3 with USP39 revealed by VIP assay.
(a) Schematic protocol of a visible immunoprecipitation (VIP) assay. HEK293T cells were transfected with expression vectors for Grainyhead-like 3 (GRHL3) protein fused to enhanced green fluorescent protein (EGFP) (*EGFP-Grhl3*) and ubiquitin-specific protease 39 (USP39) fused to red fluorescent protein (*RFP-Usp39*), respectively (a; left top). After the expression of fluorescent fusion proteins was shown under a fluorescence microscope (a; left bottom), cell lysates were prepared and processed for immunoprecipitation with glutathione *S*-transferase (GST)-tagged anti-GFP–nanobody pre-bound to glutathione-sepharose-4B beads. Beads bearing immunoprecipitates were directly observed with a fluorescence microscope. A GRHL3-fused EGFP protein interacted with a USP39-fused RFP protein. Both green and red signals are detected on the surface perimeter of the beads (a; right upper panel). (b–d) For EGFP-GRHL3 protein only, a green but not red signal was detected (b). For RFP-USP39 protein only, neither green nor red signals were detected (c). Red signals were detected on the surface of EGFP-positive nanobody beads (d). (e) Red fluorescent protein fluorescence intensities in the acquired images were measured with a Fluoview FV3000 confocal laser scanning microscope (Olympus) using cellSens software. Scale bars represent 100 μm (a), 200 μm (b–d).

Figure S4. Live imaging of *halo-tag Usp39* and expression patterns of each sub-domain of *Grhl3* fused to EGFP and endogenous USP39 protein in MCF7 cells.
(a) Subcellular localization of ubiquitin-specific protease 39 (USP39) in MCF7 cells. MCF7 cells transfected with a *HaloTag-Usp39* cDNA plasmid were incubated in medium containing tetramethylrhodamine (TMR)-ligand (red), Hoechst (blue, nucleus), and wheat germ agglutinin (WGA, green, cytoplasm). (b–f) Subcellular localization of four

types of Grainyhead-like 3 (GRHL3) products: full-length (b), transactivation (TA) domain (c), CP2 domain for DNA binding (d)[2], and ubiquitin (Ub)-like holding domain (e,f)[3] fused with enhanced green fluorescent protein (EGFP), respectively [1], and transfected into MCF7 cells. EGFP (green), USP39 (magenta), and 4,6'-diamidino-2-phenylindole (DAPI; nuclei, blue), respectively. Full-length GRHL3 fused to EGFP protein was mainly localized in the nucleus but not cytoplasm of MCF7 cells (b; green). Endogenous USP39 expression was found in the cytoplasm but did not overlap with nuclear GRHL3 localization (b). The TA-domain of GRHL3 fused to EGFP localized in the cytoplasm (c; green), and did not overlap with USP39 protein (c). Both EGFP-fused CP2 and Ub-like domains were distributed in both the nucleus and cytoplasm (d,e; arrowheads). Additionally, these EGFP localizations partly overlapped with endogenous USP39 in the cytoplasm (d,e). The Ub-like GRHL3 fused with EGFP localized mainly in the nucleus after transfection with *USP39* small interfering (si)RNA (f). Scale bars represent 20 μm (a–f).

Figure S5. Expression of molecular markers in *Usp39$^{-/-}$* embryos.
(a–e') Whole-mount *in situ* hybridization in wild-type (a–e) and *Usp39$^{-/-}$* (a'–e') embryos at E6.5. *Hex*, *Lim1,* and *Fgf8* transcript expression was detected in the anterior visceral endoderm in *Usp39$^{-/-}$* embryos (a'–c'; arrowheads), as well as in the wild type (a–c; arrowheads). Expression of the epiblast marker, *Oct3/4,* in the *Usp39$^{-/-}$* embryo (d') appears to be similar to that of the wild-type embryo (d). Expression of *Vangl2*, one of the core planar cell polarity (PCP) components, appears to be unchanged in the *Usp39$^{-/-}$* embryo (e') as compared with that of the wild-type embryo (e). Scale bars represent 100 μm (a–e').

Figure S6. Length of nucleus in wild-type and *Usp39$^{-/-}$* embryos.
The length of the nucleus was measured along its vertical axis at right angles in reference to the basement membrane and along the horizontal axis in parallel. Wild-type (n=4) and *Usp39$^{-/-}$* (n=6) embryos. Approximately 20 nuclei in the epiblast were measured per embryo.

Figure S7. *Usp39* is not essential for establishing hair cell polarity in cochlea development.
(a–e) Scanning electron micrographs of the basal turn of the organ of Corti from wild-type (a), *Vangl2$^{Lp/+}$* (b), *Usp39$^{+/-}$* (c), *Vangl2$^{Lp/Lp}$* (d), and *Vangl2$^{Lp/+}$; Usp39$^{+/-}$* (e) cochleae at E18.5. (f–j) Whole-mount cochlea stained for F-actin (green) and α-tubulin (red) at E18.5. Wild-type (f), *Vangl2$^{Lp/+}$* (g), *Usp39$^{+/-}$* (h), *Vangl2$^{Lp/Lp}$* (i), and *Vangl2$^{Lp/+}$; Usp39$^{+/-}$* (j) cochleae. The *Vangl2$^{Lp/Lp}$* cochlea displayed an aberrant stereocilia bundle orientation in OHCs and IHCs (d, i), while the *Vangl2$^{Lp/+}$; Usp39$^{+/-}$* cochlea was consistently and correctly oriented along the mediolateral axis (e, j). Abbreviations: IHCs, inner hair cells; OHCs, outer hair cells. Scale bars represent 10 μm (a–e) and 20 μm (f–j).

Figure S8. Expression of *Hoxd12* during limb development.
(a–g) Whole-mount *in situ* hybridization of *Hoxd12* transcripts in wild-type (a–c), *Vangl2$^{Lp/Lp}$* (d, e), and *Vangl2$^{Lp/Lp}$; Usp39$^{+/-}$* (f, g) embryos at E8.5 (a), E9.25 (b, d–g), and E10.5 (c). *Hoxd12* transcripts were first detected in the hindlimb region of the wild-type embryo at E10.5 (c; white arrowheads). In *Vangl2$^{Lp/Lp}$; Usp39$^{+/-}$* embryos, *Hoxd12* expression in the prospective hindlimb region was precociously induced (f, g; arrowheads). Scale bars represent 50 μm (a, b, d, f) and 100 μm (c, e, g).

Figure S9. Marker analysis in *β-catenin$^{-/-}$*, *Usp39$^{-/-}$*, and *Usp39$^{-/-}$;β-catenin$^{+/-}$* embryos.
(a–l) Whole-mount *in situ* hybridization analysis of wild-type (a,d,g,j), *β-catenin$^{-/-}$* (b,c,e,f), *Usp39$^{-/-}$* (h,k) and *Usp39$^{-/-}$; β-catenin$^{+/-}$* (i,l) embryos at E6.5. *Hex* (a–c,g–i) and *Cer-l* (d–f, j–l). *Hex* and *Cer-l* expressions are found in the anterior visceral endoderm in wild-type embryos (a,d,g,j; arrowheads), but remain in the distal visceral endoderm or are lost in *β-catenin$^{-/-}$* embryos (b,c,e,f; arrowheads). The phenotypes of *β-catenin$^{-/-}$* embryos are more severe than those of *Usp39$^{-/-}$* embryos, which were expressed in the anterior visceral endoderm (AVE) region (h,k). In *Usp39$^{-/-}$; β-catenin$^{+/-}$* embryos, AVE markers were expressed normally, as well as *Usp39$^{-/-}$* embryos (i,l; arrowheads). Scale bars represent 100 μm (a–l).

Figure S10. *Grhl3$^{+/-}$; Usp39$^{+/-}$* mice display retardation of hair growth at 2 weeks of age.
(a, b) Wild-type (left) and *Grhl3$^{+/-}$; Usp39$^{+/-}$* (right) mice 2 weeks after birth. The *Grhl3$^{+/-}$; Usp39$^{+/-}$* mouse displays temporary alopecia (right). (c, d) Histological examination of wild-type (c) and *Grhl3$^{+/-}$; Usp39$^{+/-}$* (d) embryos at 2 weeks of age indicated distortions in hair follicles, such as epidermal hyperplasia, sebaceous gland enlargement, and cyst formation (d). Scale bars represent 200 μm (c, d).

Figure S11. Defective leading-edge formation in *Grhl3$^{-/-}$* and *Grhl3$^{-/-}$; Usp39$^{+/-}$* embryos and co-localization of GRHL3 and USP39 during eyelid closure at E15.5.
(a–i) Scanning electron microscopy (SEM) of the eyelid region of wild-type (a–c), *Grhl3$^{-/-}$* (d–f), and *Grhl3$^{-/-}$; Usp39$^{+/-}$* (g–i) embryos at E15.5. Higher magnification views of the corresponding boxed regions in panels (a, d, g). SEM observation of E15.5 normal eyes indicates that rounded periderm cells are present in clumps all around the eyelid

margin (a–c). In *Grhl3*$^{-/-}$ and *Grhl3*$^{-/-}$*; Usp39*$^{+/-}$ typical peridermal clumps are not seen (d–i). (j–o) F-actin fibers accumulated in leading-edge cells in a wild-type embryo (j,k). F-actin expression is greatly reduced at the eyelid margin and epithelial leading edge in *Grhl3*$^{-/-}$ (l,m) and *Grhl3*$^{-/-}$*; Usp39*$^{+/-}$ embryos (n,o) at E15.5. (p–q") Merged image of *Grainyhead-like 3* (GRHL3; β-GAL; green) and ubiquitin-specific protease 39 (USP39) proteins (magenta) demonstrating a large overlap in the open (p–p"; arrows) and closed (q–q", arrowheads; flattened peridermal cells) eyelids of *Grhl3*$^{+/-}$ embryos at E15.5. Arrows indicate a regular accumulation of epithelial cells, with future periderm cells lined up on the eyelid margin (p). (r–s") Merged image of GRHL3 (r,s; green) and USP39 (r',s'; magenta) proteins demonstrating co-expression in the cytoplasm of the leading edge of eyelid cells at E15.5 of the wild type. 4,6'-diamidino-2-phenylindole (DAPI; blue). (l–n'). fp; flattened peridermal cells. Scale bars represent 20 μm (s-s"), 50 μm (b,c,e,f,h,i,r–r") and 200 μm (j–q").

Figure S12. Chemical inhibitors of the PCP pathway prevent eyelid closure of *Vangl2; Usp39* compound mutant explants *in vitro*.
(a–h) Outlook (a–b") and histological views with hematoxylin–eosin staining (c–h) of eyelid closure in explant cultures (an arrow in d; peridermal epithelial cells). Most eyelid explants from wild-type embryos at E15.25 were able to close within 24 h of culture (b,e,f). Open eyelids were further classified into two classes: middle (neither completely opened nor closed; b',g,h) or opened (completely opened eyelid; b"). (i) Classification of eyelid closure phenotypes after culture from wild-type, *Vangl2*$^{Lp/+}$, *Usp39*$^{+/-}$, and *Vangl2*$^{Lp/+}$*; Usp39*$^{+/-}$ embryos with or without chemical inhibitors of the planar cell polarity (PCP) signal (Y27632; ROCK inhibitor, NSC23766; Rac inhibitor). Scale bars represent 300 μm (c, e, g), 100 μm (d, f, h)

Figure S13. *Usp39* genetically interacts with *Grhl3*^{NLS} in neural tube closure defects.
(a) A schematic representation of *Grhl3* wild-type and *Grhl3*^{NLS} alleles. (b–e) Localization of nucleo-grainyhead-like 3 (GRHL3) protein in *Grhl3*$^{NLS/NLS}$ mutant embryos. Immunohistochemistry of GRHL3 (anti-GRHL3 ab; aa478-493, green) and 4,6'-diamidino-2-phenylindole (DAPI; magenta). GRHL3 protein was mostly expressed in the nuclei of surface ectoderm cells of a *Grhl3*$^{NLS/NLS}$ embryo (d,e). (f,g) The *Grhl3*$^{NLS/NLS}$; *Usp39*$^{+/-}$ embryo has a larger area of neural tube defects than the *Grhl3*$^{NLS/NLS}$ embryo. In this experiment, the angle of the neural tube closure was measured from the belly button in *Grhl3*$^{NLS/NLS}$ (f) and *Grhl3*$^{NLS/NLS}$*; Usp39*$^{+/-}$ (g) embryos at E15.5. (h) Quantification of angle in *Grhl3*$^{NLS/NLS}$ and *Grhl3*$^{NLS/NLS}$*; Usp39*$^{+/-}$ embryos. The *p*-value was calculated using Student's *t*-test (p=0.0055< p=0.01). Scale bars represent 20μm (b–e).

Figure S14. Generation and characterization of *Ube1* mutant mice.
(a) *Usp39*-dependent RNA expression of planar cell polarity (PCP)-related genes identified with RNA sequencing. A list shows downregulated genes in *Usp39*-deficient embryos compared with control wild-type embryos, with fold changes of the transcript level for each PCP pathway-related gene as indicated. (b) Schematic proposed models of biochemical processes involving ubiquitination (UBE1) and deubiquitination (ubiquitin-specific protease 39 [USP39]). (c) Diagrammatic representation of the *Ube1* wild-type allele and its mutant allele generated with a CRISPR/Cas9 system. A mutant allele is considered to be frameshifted null allele. (d,e) UBE1 protein expression in wild-type (d) and *Ube*$^{-}$ (e) blastocysts. UBE1 expression is observed in the cytoplasm of the outer trophectoderm layer of the wild-type blastocyst (d) but reduced in the male *Ube*$^{-}$ blastocyst (e). UBE1 (magenta) and 4,6'-diamidino-2-phenylindole (DAPI; blue). (f–g') Phalloidin (F-actin) staining of wild-type (f) and male *Ube1*$^{-}$ (g,g') blastocysts at E3.5. Male *Ube1*$^{-}$ embryos display morphological abnormalities in terms of cellular architecture (g,g'). Phalloidin (green) and DAPI (blue). (h,i) Poly-ubiquitin expression is severely decreased in male *Ube1*$^{-}$ (i), compared to wild-type (h) embryos at E4.0. (j–m) Histological views of E5.5 embryos with uterine tissues. Examples of normal (j,k) and abnormal (l,m) embryos derived from intercrossing an *Ube*$^{+/-}$ female and wild-type male. The absorbed embryo does not contain any evident embryonic structure (l,m). (n) Number of embryos obtained for each genotype: *Usp39*, *Ube1,* and compound mutant embryos at E7.5. Absorbed embryos are considered to include male embryos having three genotypes; *Ube*$^{-}$*; Usp39*$^{+/-}$ (12.5% predicted), *Ube*$^{-}$*; Usp39*$^{+/+}$ (6.25% predicted), and *Ube*$^{-}$*; Usp39*$^{-/-}$ (6.25% predicted). Scale bars represent 50 μm (d–i, m), 100 μm (k) and 200 μm (j,l).

Figure S15. Uncropped blots of Figure S2.

1. Kimura-Yoshida, C., Mochida, K., Nakaya, M.A., Mizutani, T. & Matsuo, I. Cytoplasmic localization of GRHL3 upon epidermal differentiation triggers cell shape change for epithelial morphogenesis. *Nature communications* **9**, 4059 (2018).
2. Wang, S. & Samakovlis, C. Grainy head and its target genes in epithelial morphogenesis and wound healing. *Curr Top Dev Biol* **98**, 35-63 (2012).
3. Kokoszynska, K., Ostrowski, J., Rychlewski, L. & Wyrwicz, L.S. The fold recognition of CP2 transcription factors gives new insights into the function and evolution of tumor suppressor protein p53. *Cell cycle (Georgetown, Tex.)* **7**, 2907-2915 (2008).

Table S1 Lists of antibodies, bioconjugates, oligonucleotides and cell lines

Antibody/Bioconjugate		Manufacture	Catalog No.	Source Type	conc.
Primary antibodies	active-β-catenin (anti-ABC) clone 8E7	upstate	05-665	Mouse IgG1κ	x100
	anti-acetylated tublin (clone.6-11B-1)	Sigma-Aldrich	T6793	Mouse IgG2b	x100
	β-GAL	abcam	ab9361	Chick IgY	x500
	E-CADHERIN	TAKARA	M-108	Rat IgG2a	x100
	EGFP	abcam	ab13970	Chick IgY	x500
	GRHL3 (aa195-211)	MBL (contract)		Rabbit IgG	x100
	GRHL3 (aa478-493)	Sigma (contract)		Rabbit IgG	x100
	GRHL3	Santa Cruz Biotechnology	sc-398838	Mouse IgG2bk	x100
	GST	abcam	ab181652	Goat IgG	
	human BRCHYURY	R&D System	AF2085	Goat IgG	x100
	KERATIN 17/19	Cell Signaling Technology	#3984	Rabbit IgG	x100
	N-CADHERIN	BD Transduction Labratories	610920	Mouse IgG1	x100
	anti-multi Ub	MBL	D058-3	Mouse IgG1k	x100
	PKCζ	Santa Cruz Biotechnology	sc-216	Rabbit IgG	x300
	pMLC(Ser19)	Cell Signaling Technology	3671	Rabbit IgG	x100
	PRICKLE1 (F-5)	Santa Cruz Biotechnology	sc-393034	Mouse IgG	x100
	SCRIB	Thermo Fisher	PA5-28628	Rabbit IgG	x100
	TBR-2/EOMES	abcam	ab23345	Rabbit IgG	x500
	TFAP2A	Cell Signaling Technology	#3208	Rabbit IgG	x100
	TFAP2B	Cell Signaling Technology	#2509	Rabbit IgG	x100
	TROMAI (Keratin-8)	Developmental Studies Hybridoma Bank	(TROMAIc)	Rat IgG	x300
	SCRIB	Invitrogen	PA5-28628	Rabbit IgG	x100
	Ube1 (2G2)	Santa Cruz Biotechnology	sc-53555	Mouse IgG	x100
	USP39	Sigma	AV38825	Rabbit IgG	x100
			U0385	Rabbit IgG	x100
	VANGL2	Santa Cruz Biotechnology	sc-67136	Rabbit IgG	x100
2nd antibodies	Alexa 488-conjugated rabbit anti-goat IgG	Thermo Fisher Scientific	A-11078		x200
	Alexa 488-conjugated goat anti-mouse IgG1		A-21121		
	Alexa 488-conjugated goat anti-rat IgG		A-11006		
	Alexa 568-conjugated goat anti-rabbit IgG		A-11036		
	Alexa 568-conjugated rabbit anti-goat IgG		A-11079		

**Bio-conjugates**	phalloidin	Thermo Fisher Scientific	A12379 (Alexa488)
			A12380 (Alexa568)
	HaloTag TMR ligand	Promega	G8251
	DAPI	Lonza	PA-3013

gene name	Oligonucleotides for genotyping		Enzyme digest
Grhl3-null and *Grhl3NLS* alleles	*Grhl3-cre-for*	*AATTAAGAGACGAGTGGTCAGCAGCGCCTG*	(-)
	Grhl3-cre mut	*GCAGCCCGGACCGACGATGAAGCATGTTTA*	
	Grhl3-cre wt rev	*ACCCTTACAAATTGCCGTGTGAATCCGGGC*	
Ube1 -null allele	*MUbe1_926F*	*TCCAGAAGGTCTTTAAGAGAGGCCTGGAGGACCAG*	*Xho* I
	MUbe1_1305R	*CAGCACTAATGTCACACGAGGAGAGGTGAGCACAC*	
Usp39 -null allele	*Usp39 for1*	*GCCTGTGAAAGGTTCCTATTTAAGCCGTGA*	*Bsl* I
	Usp39 rev2	*TGGGCATCTCAGACTTCAAGAAGGGGAAGC*	
Vangle2LP allele	*Vangl2Alu-for1*	*CAACAGTATCTTCTCCCTTCCTCAGGCCT*	*Hpy* 166II
	Hpy166 II rev1	*TCCTCAGAGAGTTTGAAGAAGGGCACCTTC*	
β-catenin-null allele	*RM41 internal*	AAGTTGTTTGTACAGAGTGTGGAGTTACTA	(-)
	RM42 internal	CTCTCTGCCCAAGTGTAAACTTATGAGGCC	
	RM43 internal	GGTATGTACAACATTGTTGGAACTTAGACA	

Oligonucleotides for plasmid construction	
mGRHL3-SalI	*ATGTCGACATGTCGAATGAACTTGATTTCAGGTCT*
mGRHL3-150P-NotI	*ATGCGGCCGCATGGGGGGACGGTATTGGTCTTGCC*
mGRHL3-151G-XhoI	*ATCTCGAGGGTCCGAGTAAACTGGAAGCCAGCTCC*

mGRHL3-490K-NotI	*ATGCGGCCGCACTTCAGAGGCAGCCTGTCAGAGCT*
mGRHL3-491R-XhoI	*ATCTCGAGCGAACCTGCTCACCCTTTGCTGAGGAG*
mGRHL3-NotI	*ATGCGGCCGCACATAGCTCCTTCAGGATGATCTGG*
USP39-XhoI	*ATCTCGAGATGTCCGGCCGGTCTAAGCGGGAGTCT*
USP39-204N-NotI	*ATGCGGCCGCAGTTTGCAATTTGCTGCTTTGTGAA*
USP39-205L-XhoI	*ATCTCGAGTTGGACAAGCAAGCCAAATTGTCCCGG*
USP-100R-XhoI	*ATCTCGAGCGGAGGAGCCGCCACTGCCCGTACCTG*
USP39 NotI	*ATGCGGCCGCATCAAGCCCCCTGCTGGTTGGTTTC*
mHoxd12 for	*GAATTCATCAACCGGCAGAAGCGTAAGGAA*
mHoxd12 rev	*GAATTCACTGGGAATGAGGCTGGACAGATT*

**Cell lines**	**Sources**
MCF7	RIKEN BioResource Research Center and originally deposited from Cell Resource Center for Biomedical Research Cell Bank, Tohoku University.
RT4	European Collection of Authenticated Cell Cultures (ECACC)
NIH3T3	Japanese Collection of Research Bioresources (JCRB)
HEK293	
MBT2	
G4-ES	from Dr. Andras Nagy (Lunenfeld-Tanenbaum Reseach Institute at Mount Sinai Hospital in Tronto)

Table S2 **Number of replicates and samples for experiments.**

Figure	Experiment	Lipofectioned plasmids, siRNA and chemical reagents	antibodies	cell line	replicates
Fig.1c	IHC	*mKG_N & mKG_C*		MCF7	2
Fig.1d		*mKG_N_mGrhl3 & mKG_C*			2
Fig.1e		*mKG_N & mKG_C_mUsp39*			2
Fig.1f,g		*mKG_N_mGrhl3 & mKG_C_mUsp39*			2
Fig.1h	IHC	*mKG_N & mKG_C*		NIH3T3	2
Fig.1i		*mKG_N_mGrhl3 & mKG_C*			2
Fig.1j		*mKG_N & mKG_C_mUsp39*			2
Fig.1k,l		*mKG_N_mGrhl3 & mKG_C_mUsp39*			2
Fig.2a,b	IHC		USP39 ab.	RT-4	2
Fig.2c,d	IHC		USP39 ab.	MBT2	2
Fig.2e-g	IHC	*DsiUSP39 13.1 and DsiUSP39 13.2 **	USP39 ab.	RT-4	2
Fig.2h-m	IHC	*pUC19/CAG-EGFP-mGrhl3 full length* plus *DsiRNA USP39 13.1 and 13.2*		RT-4	2
Fig.2n-s	IHC	*pUC19/CAG-EGFP-mGrhl3Ub-like domain* plus *DsiRNA USP39 13.1 and 13.2*		RT-4	2
Fig.3a	IHC	*pUC19/CAG-mGrhl3*	USP39 ab./TROMAI ab. /phalloidin	EB	2
Fig.3b	IHC	*pUC19/CAG-mGrhl3*	TROMAI ab./phalloidin	EB	>10
Fig.3c		*pUC19/CAG-mGrhl3* plus *Dsi mUsp39*			4
Fig.3d,e	IHC	*pCU19/CAG-β-catenin S37A.*	TROMAI ab./phalloidin	EB	3
Fig.3f		*pUC19/CAG-RFP-Usp39*			2
Fig.3g,h		*pCU19/CAG-β-catenin S37A / pUC19/CAG-RFP-Usp39*			3
Fig.3i	IHC	*pCU19/CAG-β-catenin S37A / pUC19/CAG-RFP-Usp39*	TFAP2A ab./ TROMAI ab.	EB	2
Fig.3j			TFAP2B ab./ TROMAI ab.		2
Fig.3k			KRT17/19 ab./ TROMAI		2
Fig.3l			SCRIB ab./ TROMAI ab.		2
Fig.3m			pMLC ab. /TROMAI ab		2
Fig.3n	IHC	*pUC/pgk-neo* , Wnt agonist	TROMAI ab./phalloidin	EB	3
Fig.3o,p		*pUC19/CAG-RFP-Usp39,* Wnt agonist			3
Fig.8a-c	IHC		GRHL3 ab./ USP39 ab.	MBT2	2
Fig.8f.g	Duolink (PLA)		GRHL3 (mouse) / USP39 (rabbit)	MBT2	2
Fig.S1d	IHC		GRHL3 aa195-211	MCF7	2
Fig.S1e		*siRNA Grhl3* (ID33752)			1
Fig.S1f		*siRNA Grhl3* (ID33754)			1
Fig.S1g			GRHL3 aa478-493		2
Fig.S1h		*siRNA Grhl3* (ID33752)			1
Fig.S1i		*siRNA Grhl3* (ID33754)			1
Fig.S1j		*pUC19/CAG-NLS-mGrhl3*			1
Fig.S4a	IHC (live imaging)	*pFN21A/halotg-hUSP39*	halotag ab (TMR ligand)	MCF7	2
Fig.S4b	IHC	*pUC19/CAG-EGFP-mGrhl3 full length*	EGFP ab./USP39 ab.	MCF7	3
Fig.S4c		*pUC19/CAG-EGFP-mGrhl3 TA domain*			3
Fig.S4d		*pUC19/CAG-EGFP-mGrhl3 CP2 domain*			3
Fig.S4e		*pUC19/CAG-EGFP-mGrhl3 Ub-like domain*			3
Fig.S4f		*pUC19/CAG-EGFP-mGrhl3 Ub-like domain plus siRNA Usp39*			2
Fig.S13g,h	IHC		PRICKLE1	MCF7	1
Fig.S13i,j		*DsiRNA USP39 13.2*			1

Figure	Experiment	Mouse genotype	antibodies / probe	Embryonic day	N=
Fig.4b	IHC	wild type	USP39 ab.	E6.5	4
Fig.4b'		*Usp39 -/-* F0-8			5
Fig.4c	HE staining	wild type		E6.5	2
Fig.4d.e		wild type			3
Fig.4f		*Usp39 -/-* F0-8			2
Fig.4g,h		*Usp39 -/-* F0-8			2
Fig.4i	IHC	wild type	EOMES. ab	E6.5	3
Fig.4i'		*Usp39 -/-* F0-8			3
Fig.4j	IHC	wild type	BRACHYURY. ab	E6.5	3

Fig.4j'	IHC	*Usp39 -/-* F0-8	BRACHYURY. ab.	E6.5	1
Fig.4k	IHC	wild type	E-CADHERIN ab./ N-CADHERIN ab.	E6.5	3
Fig.4k'		*Usp39 -/-* F0-8			4
Fig.4l	IHC	wild type	VANGL2 ab.	E6.5	3
Fig.4l'		*Usp39 -/-* F0-8			3
Fig.4m	IHC	wild type	SCRIB ab	E6.5	4
Fig.4m'		*Usp39 -/-* F0-8			2
Fig.4n	IHC	wild type	β-CATENIN ab./ F-actin	E6.5	3
Fig.4n'		*Usp39 -/-* F0-8			3
Fig.5a	IHC	wild type	PRICKLE1 ab	E6.5	5
Fig.5b		*Usp39 -/-* F0-8			3
Fig.5d,e	IHC	wild type	Phalloidin	E5.5	4
Fig.5f,g		*Usp39 -/-* F0-8			6
Fig.5h,i		wild type			3
Fig.5j,k		*Usp39 -/-* F0-8			3
Fig.5l	IHC	wild type	acetylated tublin ab.	E6.5	4
Fig.5m		*Usp39 -/-* F0-8			5
Fig.5n	IHC	wild type	PKCζ ab.	E6.5	4
Fig.5o		*Usp39 -/-* F0-8			6
Fig.6e	W-ISH	wild type	*Fgf8* probe	E9.5	16
Fig.6e'		*Vangl2Lp/Lp*			12
Fig.6e''		*Vangl2Lp/Lp; Usp39+/-*			14
Fig.7b	SEM	wild type		E17.5	2
Fig.7b'		*Grhl3-/-*			3
Fig.7c	HE staining	wild type		E17.5	2
Fig.7c'		*Grhl3-/-*			4
Fig.7e	HE staining	wild type		E15.5	2
Fig.7f	S-ISH	wild type	*Usp39 / Grhl3* probes		3
Fig.7g		wild type			3
Fig.7h-p	IHC	wild type	USP39 ab./ GTHL3 ab	E15.5	3
Fig.8h,i	Duolink (PLA)	wild type		E15.5	2
Fig.8j,k		wild type	Grhl3 (mouse)/ USP39 (rabbit)		2
Fig.8l	IHC	wild type	VANGL2 ab.	E15.5	4
Fig.8l'		*Grhl3-/-;Usp39+/-*			3
Fig.8m	IHC	wild type	CELSERI ab.	E15.5	2
Fig.8m'		*Grhl3-/-;Usp39+/-*			2
Fig.8n	IHC	wild type	pMLC ab.	E15.5	4
Fig.8n'		*Grhl3-/-;Usp39+/-*			3
Fig.9a	IHC	wild type	EOMES. Ab	E6.5	6
Fig.9b		*Usp39 -/-*			7
Fig.9c		*Ube1+/-; Usp39-/-*			4
Fig.9d	W-ISH	wild type	*Hex* probe	E7.5	4
Fig.9e		*Usp39 -/-*			8
Fig.9f		*Ube1+/-; Usp39-/-*			4
Fig.9g,j		wild type	*Foxa2* probe		4
Fig.9h,k		*Usp39 -/-*			6
Fig.9i,l		*Ube1+/-; Usp39-/-*			3
Fig.9n	IHC	wild type	PRICKLE1 ab	E6.5	3
Fig.S5a	W-ISH	wild type	*Hex* probe	E6.5	7
Fig.S5a'		*Usp39 -/-* F0-8			3
Fig.S5b	W-ISH	wild type	*Lim1* probe	E6.5	4
Fig.S5b'		*Usp39 -/-* F0-8			3
Fig.S5c	W-ISH	wild type	*Fgf8* probe	E6.5	8
Fig.S5c'		*Usp39 -/-* F0-8			2
Fig.S5d	W-ISH	wild type	*Oct3/4* probe	E6.5	4
Fig.S5d'		*Usp39 -/-* F0-8			3
Fig.S5e	W-ISH	wild type	*Vangl2* probe	E6.5	4
Fig.S5e'		*Usp39 -/-* F0-8			4
Fig.S7a	SEM	wild type		E18.5	6
Fig.S7b		*Vangl2Lp/+*			3
Fig.S7c		*Usp39+/-*			4
Fig.S7d		*Vangl2Lp/Lp*			4
Fig.S7e		*Vangl2Lp/+; Usp39+/-*			3
Fig.S7f	IHC	wild type	phalloidin; tublin ab.	E18.5	3
Fig.S7g		*Vangl2Lp/+*			3
Fig.S7h		*Usp39+/-*			3
Fig.S7i		*Vangl2Lp/Lp*			4
Fig.S7j		*Vangl2Lp/+; Usp39+/-*			3
Fig.S8a				E8.5	2

Fig.S8b	W-ISH	wild type	*Hoxd12* probe	E9.25	7
Fig.S8c				E10.5	1
Fig.S8d,e		*Vangl2Lp/Lp*		E9.25	7
Fig.S8f,g		*Vangl2Lp/Lp; Usp39+/-*		E9.25	7
Fig.S9a	W-ISH	wild type	*Hex* probe	E6.5	3
Fig.S9b,c		*β-catenin -/-*			4
Fig.S9d	W-ISH	wild type	*Cer-l* probe	E6.5	4
Fig.S9e,f		*β-catenin -/-*			3
Fig.S9g	W-ISH	wild type	*Hex* probe	E6.5	3
Fig.S9h		*β-catenin -/-*			4
Fig.S9i		*Usp39 -/- ; β-catenin -/-*			5
Fig.S9j	W-ISH	wild type	*Cer-l* probe	E6.5	3
Fig.S9k		*β-catenin -/-*			8
Fig.S9l		*Usp39 -/- ; β-catenin -/-*			4
Fig.S10c	HE staining	wild type		P14	2
Fig.S10d		*Grhl3+/-;Usp39+/-*			3
Fig.S11a-c	SEM	wild type		E15.5	5
Fig.S11d-f		*Grhl3-/-*			4
Fig.S11g-i		*Grhl3-/-;Usp39+/-*			2
Fig.S11j,k	IHC	wild type	F-actin	E15.5	4
Fig.S11l,m		*Grhl3-/-*			3
Fig.S11n,o		*Grhl3-/-;Usp39+/-*			3
Fig.S11p-q"	IHC	*Grhl3+/-*	β-GAL ab. / USP39 ab.	E15.5	3
Fig.S11r-s"	IHC	wild type	USP39 ab./ GTHL3 ab	E15.5	3
Fig.S12c,d	HE staining	wild type (no culture)		E15.25	3
Fig.S12e-h		wild type (24 hrs culture)			4
Fig.S12i	Bouin's Fixative	wild type (no chemical reagent)		E15.25	34
		wild type (Y27632)			36
		wild type (NSC23766)			24
		Vangl2Lp/+ (no chemical reagent)			34
		Vangl2Lp/+ (Y27632)			32
		Vangl2Lp/+ (NSC23766)			18
		Usp39+/- (no chemical reagent)			28
		Usp39+/- (Y27632)			18
		Usp39+/- (NSC23766)			12
		Vangl2Lp/+; Usp39+/- (no chemical reagent)			26
		Vangl2Lp/+; Usp39+/- (Y27632)			30
		Vangl2Lp/+; Usp39+/- (NSC23766)			20
Fig.S13b,c	IHC	wild type	GRHL3 ab.	E8.5	3
Fig.S13d,e		*Grhl3NLS/NLS*			3
Fig.S13f	IHC	wild type		E15.75	6
Fig.S13g		*Grhl3NLS/NLS*			11
Fig.S14d	IHC	wild type	UBE1 ab.	E3.5	3
Fig.S14e		*Ube1-* (male)			3
Fig.S14f	IHC	wild type	Phalloidin	E3.5	5
Fig.S14g,g'		*Ube1-* (male)			1
Fig.S14h	IHC	wild type	poly-Ub	E3.75	6
Fig.S14i		*Ube1- (male)*			6
Fig.S14j-k	HE staining	wild type		E5.5	3
Fig.S14l-m,		*Ube1- (male)*			4

* conc. 0.1 nM, 1 nM, 10 nM

Supplementary Methods

GST-GRHL3 affinity column and nanoLC/MS/MS

GST-GRHL3 was constructed in pGEX4T3 (GE Healthcare) by PCR amplification and expressed in *Escherichia coli*. GST (control) and GST-GRHL3 expressed in cell lysates were incubated with glutathione magnet beads, MagneGST Glutathione Particles (Promega; 30 µL volume) for 1 h at 4˚C, and washed three times in washing buffer A (50 mM Tris-HCl pH 7.5, 0.3M NaCl, 0.1% NP-40). Cell extracts were prepared from MCF7 cells by sonication in cell extraction buffer (50 mM HEPES pH 7.4, 0.3 M NaCl, 0.2% NP40, protease inhibitor cocktail cOmplete [Roche], Benzonase Nuclease 0.25 U [Novagen]) for 15 sec on ice. The suspension was centrifuged at 12,000g for 10 min and the supernatant was used as cell extracts. MCF7 cell extracts were incubated with GST (control) and GST-GRHL3–bound magnet beads for 12 h in the presence of 0.25U benzonase nuclease (Novagen) at 4˚C. After washing three times with washing buffer (0.15 M NaCl, 0.1% NP-40, 50 mM HEPES pH 7.4), GST (control), and GST-GRHL3 binding proteins were eluted twice in 300 µL elution buffer (1.2 M NaCl, 50 mM HEPES pH 7.4). The elutes were concentrated and desalted using Amicon ultra-4-10k (Millipore) centrifugal filter units, suspended in SDS–polyacrylamide gel electrophoresis (PAGE) sample buffer, and boiled for 5 min. The samples were resolved by SDS–PAGE. The gel was stained using a Wako Silver Stain MS Kit (Fujifilm Wako Chemicals). Gel slippage was reduced with 100 mM of dithiothreitol and alkylated by 100 mM iodoacetamide. After washing, the gel was incubated with trypsin overnight at 30°C. Recovered peptides were desalted with a ZipTips C18 column (Millipore). Samples were analyzed by nanoscale liquid chromatography tandem-mass spectrometry (nanoLC/MS/MS) systems (DiNa HPLC system Kya Technologies Corporation /QSTAR XL Applied Biosystems). Mass data acquisitions were piloted by Mascot software.

Immunoprecipitation and immunoblotting

MCF7 cells were lysed in lysis buffer (0.3 M NaCl, 0.1% NP40, 50 mM HEPES [pH7.5]) plus protease inhibitors (cOmplete [Roche]) by sonication for 15 sec on ice. After centrifugation at 18,000 ×g for 10 min, protein concentrations were measured, and equal amounts of lysates were used for immunoprecipitation. Immunoprecipitation was performed using the GRHL3 antibody (GRHL3 [C-12] Santa Cruz Biotechnology; Cat. no. sc-398838) and protein MagneGST^{TM} Glutathione Particles (Promega). Thereafter, the precipitates were washed three times in washing buffer (0.15 M NaCl, 0.1% NP40, 50 mM HEPES [pH.7.5]) and immune complexes eluted with elution buffer (1.2 M NaCl, 50 mM HEPES [pH.7.5]) for 5 min on ice. The immunoprecipitated proteins were then separated by SDS–PAGE. Western blotting was performed with the GST antibody (Table S3).

Purification of bacterially expressed recombinant GRHL3 and USP39

GST-GRHL3 and GST-USP39 expression constructs were generated by subcloning into pGEX4T3 (GE Healthcare) by PCR amplification (Table S3). GST-GRHL3 and GST-USP39 plasmids were used to transform *E. coli* (XL-10-Gold, Agilent Technologies). Cultures were grown for 12 h at 28ºC to an attenuance of 0.6 at 600 nm. Cells were then induced for 2 h at 37ºC in the presence of 1 mM isopropyl-β-D-thiogalactopyranoside. For GST-GRHL3 and GST-USP39 purifications, the washed pellets were resuspended in lysis buffer (0.3 M NaCl, 0.2% NP40, 50 mM Tris-HCl pH 7.2). After sonication, supernatants of cell lysates were incubated with glutathione Sepharose 4B (Cytiva Life Sciences; 17075601). Beads were washed twice with lysis buffer and three times with washing buffer (0.15 M NaCl, 0.2% NP-40, 50 mM Tris-HCl pH7.2).

Analysis of the organ of Corti

Temporal bones were dissected from wild-type and mutant embryos at E18.5. The surrounding cartilages were removed to expose the cochlea, and the anlage of Reissner's membrane was dissected to expose the sensory epithelium. Dissected tissues were fixed in 4% PFA at 4ºC for 1 h. To visualize the orientation of the stereocilia bundle, fixed tissues were stained with phalloidin (Molecular Probes) and the anti-acetylated tubulin antibody (Table S3).

Explant culture of mouse eyelid primordial tissue

Eyelid tissue explants were cultured as previously described [1, 2]. In brief, eyelids from embryos at E15.25 were isolated with micro-scissors under a Leica M205C stereomicroscope and cultured in 12-well plates containing 1 mL of Defined Keratinocyte-SFM (Gibco; cat.no. 10744019) at 37ºC in a humidified atmosphere of 5% CO_2 for 24 h. For planar cell polarity (PCP) inhibition experiments, eyelid tissue explants were cultured in the presence of Y27632 (0.75 µM; Nacalai; cat. no. 08945-71) and NSC23766 (3.75 µM; Chemdea LLC; cat. no. CD0192) for 24 h.

Visible immunoprecipitation assay

A visible immunoprecipitation (VIP) assay was carried out as described previously [3, 4]. Expression vectors for *EGFP-Grhl3* and *RFP-Usp39* were transfected into HEK293T cells grown in a six-well plate using Lipofectamine LTX (Invitrogen) and cultured for 3 days. Lysates were prepared from the transfected cells in 250 μL of HNTG cell lysis buffer (20 mM HEPES, pH.7.4, 150 mM NaCl, 0.1% Triton X-100, and 10% glycerol) containing a protease inhibitor cocktail (cOmplete Mini; Roche; cat.no.1836153) for 15 min on ice. The lysates were centrifuged at 13,200 rpm for 15 min at 4°C and supernatant (200 μL) was transferred to 0.5 ml tubes that contained anti-GFP nanobody bound to glutathione-Sepharose 4B beads (~5 μL bed volume) and incubated for 24 h at 4°C with constant rotation. After centrifugation at 2000 ×g for 20 sec, the precipitated beads were washed three times with lysis buffer (200 μL). The beads bearing fluorescent fusion proteins were observed under a Fluoview FV3000 fluorescent microscope (Olympus) using cellSens software. Image acquisition was performed under fixed conditions in each set of experiments.

RNA-Seq analysis

Acquired RNA-sequencing (seq) data involved wild-type and *Usp39* homozygous knockout embryos at E6.5, representing two sample groups (Wt vs *Usp39*$^{-/-}$) by three biological replicates (6~10 embryos per group) for a total of six samples. Total RNA was isolated from embryos using NucleoSpin RNA XS (Takara; cat. no. U0902S) and then amplified using a SMART-seq v4 Ultra Low Input RNA kit for Sequencing (Takara; cat. no. Z4888N) according to the manufacturer's instructions. First-stranded cDNA was synthesized with a Nextera XT DNA Sample Preparation kit (illumina; cat.no. FC-131-1024). Transcriptome sequencing (RNA-seq) was performed on 150 bases in paired-end mode by a NovaSeq system (illumina). The adapter sequences and low-quality regions were trimmed by Cutadapt v1.1 and Trimmomatic v0.32, respectively. After pre-processing, the reads were mapped to Mus musculus reference genome GRCm38 by using TopHat v2.4.0. The mapped reads to 9 target genes were extracted by Samtools v0.1.18, then, were de novo assembly using Trinity v2.4.0, respectively.

1. Tao, H. *et al.* A dual role of FGF10 in proliferation and coordinated migration of epithelial leading edge cells during mouse eyelid development. *Development (Cambridge, England)* **132**, 3217-3230 (2005).
2. Bian, G. *et al.* Sphingosine 1-phosphate stimulates eyelid closure in the developing rat by stimulating EGFR signaling. *Sci Signal* **11** (2018).
3. Katoh, Y., Nozaki, S., Hartanto, D., Miyano, R. & Nakayama, K. Architectures of multisubunit complexes revealed by a visible immunoprecipitation assay using fluorescent fusion proteins. *Journal of cell science* **128**, 2351-2362 (2015).
4. Katoh, Y. *et al.* Overall Architecture of the Intraflagellar Transport (IFT)-B Complex Containing Cluap1/IFT38 as an Essential Component of the IFT-B Peripheral Subcomplex. *J Biol Chem* **291**, 10962-10975 (2016).
