# Supplementary material for: USP39 is essential for mammalian epithelial morphogenesis through upregulation of planar cell polarity components
Source: Commun Biol. 2022 Apr 19;5:378. doi: 10.1038/s42003-022-03254-7 (PMC9018712; doi:10.1038/s42003-022-03254-7)
Supplement: Supplementary file 3 — Description of Additional Supplementary Files [file 42003_2022_3254_MOESM3_ESM.pdf]

Description of Additional Supplementary Files

File name: Supplementary Data 1
Description: RNA-sequencing data for Usp39 homozygous knockout embryos at E6.5, downregulated genes compared to wildtype

File name: Supplementary Data 2
Description: RNA-sequencing data for Usp39 homozygous knockout embryos at E6.5, upregulated genes compared to wildtype

File name: Supplementary Data 3
Description: Statistical analyses of Figures 5c, 6d, 7d, S3e, S12i, and S13h.
