# Supplementary material for: USP39 is essential for mammalian epithelial morphogenesis through upregulation of planar cell polarity components
Source: Commun Biol. 2022 Apr 19;5:378. doi: 10.1038/s42003-022-03254-7 (PMC9018712; doi:10.1038/s42003-022-03254-7)
Supplement: Supplementary file 7 — Reporting Summary [file 42003_2022_3254_MOESM7_ESM.pdf]

nature portfolio

Corresponding author(s): Isao Matsuo, Chiharu Kimura-Yoshida

Last updated by author(s): Feb 25, 2022

Reporting Summary

Nature Portfolio wishes to improve the reproducibility of the work that we publish. This form provides structure for consistency and transparency in reporting. For further information on Nature Portfolio policies, see our Editorial Policies and the Editorial Policy Checklist.

Statistics

For all statistical analyses, confirm that the following items are present in the figure legend, table legend, main text, or Methods section.

n/a	Confirmed	
☐	☒	The exact sample size (n) for each experimental group/condition, given as a discrete number and unit of measurement
☐	☒	A statement on whether measurements were taken from distinct samples or whether the same sample was measured repeatedly
☐	☒	The statistical test(s) used AND whether they are one- or two-sided<br>*Only common tests should be described solely by name; describe more complex techniques in the Methods section.*
☒	☐	A description of all covariates tested
☒	☐	A description of any assumptions or corrections, such as tests of normality and adjustment for multiple comparisons
☐	☒	A full description of the statistical parameters including central tendency (e.g. means) or other basic estimates (e.g. regression coefficient) AND variation (e.g. standard deviation) or associated estimates of uncertainty (e.g. confidence intervals)
☐	☒	For null hypothesis testing, the test statistic (e.g. F, t, r) with confidence intervals, effect sizes, degrees of freedom and P value noted<br>*Give P values as exact values whenever suitable.*
☒	☐	For Bayesian analysis, information on the choice of priors and Markov chain Monte Carlo settings
☒	☐	For hierarchical and complex designs, identification of the appropriate level for tests and full reporting of outcomes
☒	☐	Estimates of effect sizes (e.g. Cohen's d, Pearson's r), indicating how they were calculated

Our web collection on statistics for biologists contains articles on many of the points above.

Software and code

Policy information about availability of computer code

Data collection	n/a
Data analysis	n/a

For manuscripts utilizing custom algorithms or software that are central to the research but not yet described in published literature, software must be made available to editors and reviewers. We strongly encourage code deposition in a community repository (e.g. GitHub). See the Nature Portfolio guidelines for submitting code & software for further information.

Data

Policy information about availability of data

All manuscripts must include a data availability statement. This statement should provide the following information, where applicable:

- Accession codes, unique identifiers, or web links for publicly available datasets
- A description of any restrictions on data availability
- For clinical datasets or third party data, please ensure that the statement adheres to our policy

All data generated or analysed during this study are included in this published article, Figures S1 to S15, Table S1, S2 and Supplementary Data 1 to 3. RNA-seq raw data were deposited with DNA Data bank of Japan (DDBJ) .

Field-specific reporting

Please select the one below that is the best fit for your research. If you are not sure, read the appropriate sections before making your selection.

☒ Life sciences ☐ Behavioural & social sciences ☐ Ecological, evolutionary & environmental sciences

For a reference copy of the document with all sections, see nature.com/documents/nr-reporting-summary-flat.pdf

Life sciences study design

All studies must disclose on these points even when the disclosure is negative.

Sample size	In our In vitro ES cell differentiation assays, we always analyzed from 10 to 20 embryoid bodies for one experimental group.
Data exclusions	No data exclusion.
Replication	We have verified reproducibility. Each result described in the paper is based on more than two independent biological replicates in principles (see Table S2).
Randomization	For experiments were our samples were exposed to chemical inhibitors, samples were randomly allocated to control and experimental groups.
Blinding	In vitro studies we have analyzed the data blindly. Regarding the mutant embryos, most of experimental groups, genotyping was done after data collection.

Reporting for specific materials, systems and methods

We require information from authors about some types of materials, experimental systems and methods used in many studies. Here, indicate whether each material, system or method listed is relevant to your study. If you are not sure if a list item applies to your research, read the appropriate section before selecting a response.

Materials & experimental systems

n/a	Involved in the study	
☐	☒	Antibodies
☐	☒	Eukaryotic cell lines
☒	☐	Palaeontology and archaeology
☐	☒	Animals and other organisms
☒	☐	Human research participants
☒	☐	Clinical data
☒	☐	Dual use research of concern

Methods

n/a	Involved in the study	
☒	☐	ChIP-seq
☒	☐	Flow cytometry
☒	☐	MRI-based neuroimaging

Antibodies

Antibodies used	We described all antibodies, their concentrations used, catalog number and company names in Table S1.
Validation	Validation of GRHL3 and USP39 antibodies were carefully done (Figures 2,4, and S1).

Eukaryotic cell lines

Policy information about cell lines

Cell line source(s)	MCF7, RT4, MBT2, NIH3T3, HEK293, G4 ES
Authentication	MCF7 was obtained from RIKEN BioResource Research Center and originally deposited from Cell Resource Center for Biomedical Research Cell Bank, Tohoku University. RT4 cells were obtained from European Collection of Authenticated Cell Cultures （ECACC） repository through Sumitomo Dainippon Pharma Co., Ltd. NIH3T3, HEK293, and MBT2 cells were obtained from the Japanese Collection of Research Bioresources (JCRB, Japan). G4 ES cells were obtained from Dr. Andras Nagy (Lunenfeld-Tanenbaum Research Institute at Mount Sinai Hospital in Toronto).
Mycoplasma contamination	No Mycoplasm contamination.
Commonly misidentified lines (See ICLAC register)	*Name any commonly misidentified cell lines used in the study and provide a rationale for their use.*

Animals and other organisms

Policy information about studies involving animals; ARRIVE guidelines recommended for reporting animal research

Laboratory animals	We conducted animal experiments in line with ARRIVE guidelines. All mouse studies followed the fundamental guidelines for proper conduct of animal experiments and related activities in academic research institutions under the jurisdiction of the Ministry of Education, Culture, Sports, Science, and Technology of Japan and were approved by institutional committees at the Research Institute, Osaka Women's and Children's Hospital for animal and recombinant DNA experiments. These were also described in the Methods section.
Wild animals	No wild animals were used in the study.
Field-collected samples	No field-collected samples were used in the study.
Ethics oversight	Institutional committees at the Research Institute, Osaka Women's and Children's Hospital for animal and recombinant DNA experiments.

Note that full information on the approval of the study protocol must also be provided in the manuscript.
